# Artificial Intelligence for Predicting Difficult Airways: A Review

**DOI:** 10.3390/jcm14238600

**Published:** 2025-12-04

**Authors:** Meruyert Alatau, Johann Bauer, Vitaliy Sazonov

**Affiliations:** 1 Department of Medicine, School of Medicine, Nazarbayev University, Kerey Zhanibek Handar Street 5/1, Astana 010000, Kazakhstan; meruyert.alatau@nu.edu.kz; 2 Centre for Cognitive Science, Institut für Psychologie, Technische Universität, Alexanderstraße 10, 64283 Darmstadt, Germany; 3 Pediatric Anesthesiology and Intensive Care Unit, Mother and Child Health Center, University Medical Center, Turan 32, Astana 010000, Kazakhstan; 4 Department of Surgery, School of Medicine, Nazarbayev University, Kerey Zhanibek Handar Street 5/1, Astana 010000, Kazakhstan

**Keywords:** artificial intelligence, machine learning, difficult airway, airway management, intubation prediction

## Abstract

**Background**: Accurately predicting difficult airways is essential to ensuring patient safety in anesthesiology and emergency medicine. However, traditional assessment tools often lack sufficient sensitivity and specificity, particularly in high-pressure or resource-limited settings. Artificial intelligence (AI) and machine learning (ML) have emerged as promising tools for enhancing airway assessment. **Objective**: This review evaluates the performance of AI- and ML-based models for predicting difficult airways and compares them with traditional clinical methods. The review also analyzes the models’ methodological robustness, clinical applicability, and ethical considerations. **Methods**: A comprehensive literature search was conducted across PubMed, Web of Science, and Scopus to identify studies published between 2020 and 2025 that employed AI/ML models to predict difficult airways. Both original research and review articles were included. Key metrics, such as the area under the curve (AUC), sensitivity, and specificity, were extracted and compared. A qualitative analysis was performed to focus on dataset characteristics, validation strategies, model interpretability, and clinical relevance. **Results**: AI models demonstrated superior performance compared to traditional assessment tools. The MixMatch semi-supervised deep learning (DL) model achieved the highest performance (area under the curve [AUC] of 0.9435, sensitivity of 89.58%, and specificity of 90.13%). Models that used facial imaging combined with deep learning consistently outperformed those that relied solely on clinical parameters. However, methodological heterogeneity, a lack of standardized evaluation metrics, and limited population diversity impeded cross-study comparability. Few studies incorporated interpretability frameworks or addressed ethical challenges related to data privacy and algorithmic bias. **Conclusions**: AI and ML models have the potential to transform the assessment of difficult airways by improving diagnostic accuracy and enabling real-time clinical decision support.

## 1. Introduction

Airway management is a critical procedure in anesthesia, emergency medicine, and critical care. Situations involving a difficult airway, which are characterized by challenges in mask ventilation or tracheal intubation, pose significant risks and can lead to morbidity and mortality. These situations account for approximately one-third of anesthesia-related deaths [1,2]. The American Society of Anesthesiologists defines a difficult airway as a scenario in which a trained anesthesiologist has difficulty performing these essential tasks [3].

The incidence of difficult intubation ranges from 1.9% to 27%, which is substantially higher than the incidence of difficult mask ventilation, ranging from 1.4% to 5% [4,5,6]. These difficulties can lead to severe complications such as tracheal or esophageal injury, aspiration, hypoxemia, irreversible brain damage, and death [1,2]. Repeated intubation attempts in emergencies further increase the risk of complications by causing mechanical damage, such as laryngeal edema and hemorrhage, which can make subsequent interventions more difficult [7].

Traditional methods of assessing difficult airways include the Mallampati classification, thyromental distance (TMD), and the upper lip bite test (ULBT). Comprehensive evaluations, such as the modified LEMON criteria, are also used [8,9,10]. However, these bedside tests have significant limitations. They rely heavily on subjective interpretation and demonstrate moderate sensitivity and specificity. Their effectiveness also varies with patient demographics, such as age, gender, and ethnicity [11,12]. For instance, the Mallampati test has been reported to have a sensitivity of 32% and a specificity of 85%, while the modified LEMON criterion has a sensitivity of 85% and a specificity of 47% [11,13]. These limitations have led clinicians and researchers to explore advanced technological solutions, specifically artificial intelligence (AI) and machine learning (ML), to improve the accuracy of airway assessments and reduce subjectivity. AI methods, particularly deep learning (DL) and computer vision, can process large datasets, identify intricate patterns, and predict clinical outcomes with high precision [14,15]. AI applications have already demonstrated notable success in fields such as dermatology, radiology, and oncology by automating image analysis and improving diagnostic accuracy [16].

Recent studies in the field of airway management have increasingly focused on AI-based assessments using patient facial and anatomical images to predict difficult airways. These predictive models use computer vision and deep learning techniques to provide faster, more objective, and potentially more accurate assessments than traditional methods [10,17]. AI’s potential to assist clinicians without specialized airway management training further highlights its utility, particularly in emergency settings or resource-limited environments [7]. Despite promising early results, critical knowledge gaps remain. Most existing AI models have only been tested under controlled conditions and lack extensive validation across diverse patient populations. Furthermore, the absence of universally accepted evaluation criteria or standardized datasets complicates comparisons across studies. Thus, further systematic exploration and comparison of AI-based airway assessment tools are required.

A growing body of literature emphasizes the importance of explainability in clinical decision support systems (CDSSs) beyond raw predictive performance [18]. Recent meta-analyses of explainable AI (XAI) in healthcare CDSSs have shown that while black-box models can be highly accurate, they often lack transparency [19]. This lack of transparency can undermine clinician trust and impede adoption at the bedside. These studies systematically catalog XAI techniques, such as feature attribution, saliency maps, and counterfactual explanations, and demonstrate how these techniques can make model outputs more interpretable and clinically meaningful while also exposing potential failure modes and biases [20]. Incorporating similar XAI approaches into AI-based airway assessment could help bridge the gap between highly complex deep learning architectures and the practical need for understandable and defensible decisions in anesthesiology and emergency medicine [21].

This study primarily aims to review and critically analyze recent developments in AI-based predictive models for difficult airway assessment. We will compare the accuracy, sensitivity, and specificity of these models with traditional methods. Specifically, we focus on models that use facial imaging to evaluate their clinical applicability, identify the most effective approaches, and suggest future research directions to advance their integration into routine clinical practice.

## 2. Materials and Methods

This study was conducted as a systematic literature review, focusing on recent developments in artificial intelligence (AI) and machine learning (ML) models for assessing difficult airways. The review followed the PRISMA 2020 guidelines. A comprehensive literature search was performed in PubMed, Web of Science, and Scopus, which together provide extensive coverage of peer-reviewed biomedical and clinical research. The search covered studies published from January 2020 to February 2025. The search used the following keywords and their synonyms combined with Boolean operators: “artificial intelligence,” “AI,” “machine learning,” “deep learning,” “difficult airway assessment,” “difficult intubation,” and “airway management.” Boolean operators (“AND,” “OR”) were used to refine the search strategy in each database. The detailed search strings for all sources are provided in Supplementary Table S1. Preliminary scoping indicated that most records in computer science–focused repositories, such as IEEE Xplore, ACM Digital Library, and Arxiv, lacked evaluation in human clinical populations and did not report diagnostic performance metrics aligned with our predefined eligibility criteria. To maintain a clinically oriented, patient-centered synthesis, the review was restricted to biomedical indexing services.

A review protocol was developed a priori, specifying the research question, eligibility criteria, and data extraction items. The protocol was not registered in PROSPERO because the project initially evolved from a time-constrained, methods-focused narrative synthesis. We acknowledge that the lack of prospective protocol registration reduces transparency and increases the risk of unrecorded post hoc modifications. Therefore, to enhance reproducibility, the core protocol (inclusion/exclusion criteria, outcomes, and analysis plan) is provided in Supplementary Table S2.

Eligible studies were original research or review articles published in peer-reviewed, indexed journals within the last five years (2020–2025). Studies had to focus specifically on the application of artificial intelligence, machine learning, or deep learning models aimed at predicting or assessing difficult airways to be included. Furthermore, studies were required to use patient facial images or anatomical data to develop predictive AI models. Studies were excluded if they were editorials, letters, abstracts, or non-peer-reviewed publications; if they did not use patient image-based data to develop AI models; or if they were published before 2020. The initial screening involved reviewing titles and abstracts. Eligible articles were then evaluated in full to ensure adherence to these selection criteria.

**Figure 1:**
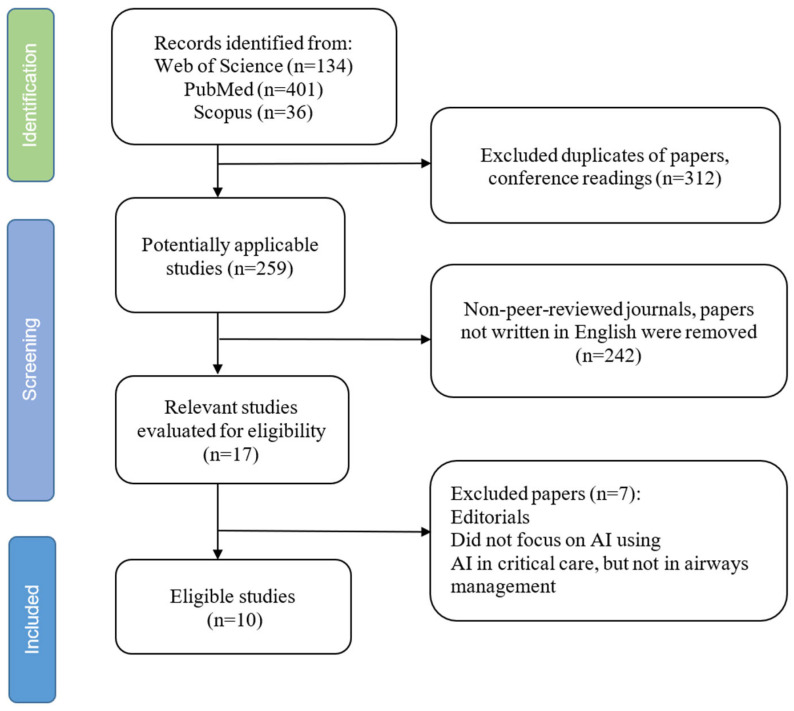
Flowchart of search strategy.

A total of 17 articles were initially identified. After applying the inclusion and exclusion criteria, one editorial and three studies published before 2020 were removed. Two additional studies from non-peer-reviewed sources and four studies that did not utilize patient image data were removed. Ultimately, ten articles met the inclusion criteria: three reviews and seven original research papers. The selection process is illustrated in Figure 1.

Data extraction included general characteristics of each study (authors, publication year, study type, and research objective), detailed information on AI methodology (type of AI model, specific algorithms used, dataset details, number of participants, and participant ethnicity), evaluation metrics (sensitivity, specificity, accuracy, area under the receiver operating characteristic curve (AUC)) and reported limitations or challenges.

For comparative analysis, studies were grouped into two main categories: review articles and original research articles. Review articles were analyzed based on the number and type of studies reviewed, the AI methods discussed, the evaluation metrics used, and the conclusions drawn. Original research articles were analyzed based on the number of participants, participant demographics, AI models used, data preprocessing techniques, and evaluation metrics.

To enhance comparability and clarity, the data were presented in tables summarizing the key findings and methodologies. Special attention was given to identifying potential biases, including those related to participant ethnicity, dataset imbalance, and methodological rigor.

The methodological quality of the original studies was critically assessed with respect to the reproducibility and transparency of the AI model development methods. Studies were evaluated based on the explicitness of the data preprocessing steps, the clarity of the algorithmic frameworks, and the availability of sufficient details for model replication. The risk of bias of the included studies was evaluated using the Quality Assessment of Diagnostic Accuracy Studies-2 (QUADAS-2) tool. QUADAS-2 assesses four critical domains: patient selection, index test (AI model), reference standard, and flow and timing. Each domain was judged as “low risk,” “high risk,” or “unclear risk.” Applicability concerns for patient selection, index test, and reference standard were similarly assessed. Two independent reviewers performed these assessments, and disagreements were resolved through discussion or by consulting a third reviewer.

As this review was based on publicly available, published studies, no ethical approval was required.

Due to the nature of the systematic review, no formal statistical analyses were performed. Instead, a narrative synthesis and descriptive comparison of the included studies were undertaken, emphasizing performance metrics of AI models such as area under the curve (AUC), sensitivity, specificity, and accuracy.

## 3. Results

Ten studies that met the inclusion criteria were analyzed, including three review articles and seven original research articles. The studies primarily used AI techniques, such as machine learning, deep learning, and computer vision models, to predict difficult airway management. A summary of these studies is provided in Table 1.

### 3.1. Review Article Analysis

The review articles analyzed provided comprehensive evaluations of existing AI models. The reviews primarily focused on evaluating AI methods, such as machine learning algorithms and deep learning architectures (e.g., convolutional and recurrent neural networks), as well as computer vision techniques, for predicting difficult airways. The reviews reported varying performance metrics and highlighted limitations, including dataset biases, limited generalizability across diverse patient populations, and variable accuracy dependent on model complexity and dataset quality. Additionally, the reviews suggested future directions, such as further validating the models across broader patient populations and enhancing the interpretability of AI models (see Table 2).

### 3.2. Original Research Articles Analysis

Seven original research articles provided detailed insights into developing and validating AI models for assessing difficult airways using patient facial and anatomical images. Table S3A,B in the Supplementary Materials present key characteristics of these studies, including patient demographics, AI techniques employed, and main performance metrics.

The studies utilized diverse datasets, with participant numbers ranging from 202 to over 5000. Participant demographics varied significantly and predominantly reflected Asian populations (Japanese, Korean, and Chinese), raising concerns about the models’ generalizability to other ethnic groups.

The results of the QUADAS-2 assessment: the overall methodological quality of the included studies was robust, with most domains judged as “low risk.” Patient selection methods, AI model transparency, and consistent use of reference standards were generally adequate across studies, indicating minimal risk of significant bias. However, minor concerns were identified in some studies, primarily related to unclear documentation of independent validation of airway assessments and variability in reference standard application, particularly in Kim et al. (2024) [22].

The AI methodologies employed ranged from standard machine learning (ML) algorithms, such as Balanced Random Forest, Logistic Regression, and Gradient Boosting, to more complex deep learning (DL) architectures, including ResNet18, EfficientNet-B5, and Convolutional Neural Networks. Performance metrics were primarily evaluated through the area under the receiver operating characteristic curve (AUC), as well as sensitivity and specificity. Considering that these original research papers invented new or further modified existing AI models, the accessibility of their methodology was believed to be important for judgment. As a result, this assessment was included in Table 3. In order to have common criteria for analyzing original research articles and review articles, comparison with existing methods, conclusions, and future perspectives were also presented (see Table 3).

The best-performing AI model was a semi-supervised deep learning approach by Wang et al. (2023) [25]. This model demonstrated high accuracy (0.9), sensitivity (0.8958), specificity (0.9013), and an AUC of 0.9435 using minimal labeled data (30%). This result indicates substantial potential for clinical applicability. Models that used a combination of facial images and clinical parameters generally outperformed models based solely on clinical or image data, suggesting the benefit of integrating multiple data types in AI assessments.

A comparative analysis of original research articles revealed significant variability in AI performance metrics. These metrics are influenced by factors such as dataset quality, image acquisition protocols (e.g., patient positioning), and the types of AI models employed. Studies using extensive and diverse image datasets with standardized capture techniques generally achieved superior outcomes.

Furthermore, deep learning models were found to consistently outperform traditional machine learning algorithms, demonstrating higher levels of accuracy, sensitivity, and specificity. This was particularly evident in models that incorporated multi-view images and leveraged advanced computer vision techniques, such as heat maps and landmark detection. Limitations highlighted across studies included potential biases from limited ethnic diversity and inconsistent image capture and processing methods.

Analysis of methodological transparency revealed varying levels of detail across studies. Most original research articles provided adequate descriptions of their methods, including data preprocessing steps, AI architecture details, and evaluation protocols, which enabled potential reproducibility. However, some studies lacked sufficient clarity about specific algorithmic parameters and preprocessing steps, which complicates reproducibility and external validation efforts.

## 4. Discussion

AI and ML are increasingly integrated into airway assessment due to their potential to outperform traditional methods, which are often subjective. Our systematic review reveals that AI-driven models for predicting difficult airways demonstrate promising performance across multiple studies, especially when facial imaging and deep learning architectures are used. While these models demonstrate notable improvements in accuracy, sensitivity, and specificity compared to traditional assessments, several methodological and clinical limitations require careful consideration.

Traditional bedside assessments, such as the Mallampati score, thyromental distance, and modified LEMON criteria, have been foundational tools for airway evaluation [28]. However, they are often criticized for having low sensitivity and high interobserver variability [29]. These limitations have driven the exploration of AI-based approaches. Our review shows that many AI models have surpassed traditional benchmarks in predictive performance.

### 4.1. Comparative Performance of AI and Traditional Models

The original studies and review articles analyzed in this study highlight consistent and promising trends. AI models, especially those using facial imaging and deep convolutional architectures, demonstrate superior accuracy, sensitivity, and specificity compared to traditional bedside assessments. The modified LEMON score, Mallampati test, ULBT, and thyromental distance demonstrated lower AUCs (often <0.70), confirming earlier observations from De Rosa et al. [17] and Chen et al. [10] that traditional methods lack precision. For instance, Wang et al.’s MixMatch model (AUC 0.9435) outperformed all traditional benchmarks, underscoring the transformative potential of AI in airway assessment.

Among the reviewed studies, Wang et al.’s [25] MixMatch semi-supervised DL model achieved the highest performance, with an area under the curve (AUC) of 0.9435, a sensitivity of 89.58%, and a specificity of 90.13%, despite using only 30% labeled data. This underscores the model’s robustness and feasibility for clinical implementation with minimal manual annotation.

Other noteworthy models include Hayasaka et al.’s [7] model, which evaluated 16 positional image combinations and reported an AUC of 0.864 for the supine-side-closed mouth-base position, along with sensitivities and specificities exceeding 80%. Similarly, Kim et al. [22] used a DL model based on smartphone-acquired images that achieved an AUC range of 0.81–0.88. Xia et al. [26] demonstrated that simplified facial models could match or outperform traditional models; their combined image-based and clinical parameter model reached an AUROC of 0.778.

In contrast, models that relied solely on clinical data, such as those developed by Kim et al. [23] and Yamanaka et al. [27], performed less robustly. The best-performing clinical parameter model (Kim et al., 2021), which used Balanced Random Forest, achieved an AUROC of 0.79; other models that used logistic regression or MLP showed reduced performance [23]. These findings support the hypothesis that incorporating image-based features, especially when enhanced with DL, is essential to achieving higher predictive performance.

### 4.2. Limitations in Methodology and Comparability

A key challenge in synthesizing evidence from different studies is the variety of evaluation metrics and inconsistent reporting formats. De Rosa et al. (2025) [17] and Matava et al. (2020) [15], for example, reported detailed area under the curve (AUC), sensitivity, and specificity values for the models they reviewed. However, Chen et al. (2024) [10] did not provide concrete performance metrics, which limited the ability to draw quantitative comparisons.

Among the original studies, Tavolara et al. (2021) [24] evaluated a convolutional neural network (CNN)-based face region feature extractor (FRFE) model with three facial alignment strategies and reported moderate area under the curve (AUC) values (~0.6465). An ensemble model using multiple-instance learning (MIL) improved performance modestly, achieving an AUC of 0.7105. Similarly, Hayasaka et al. (2021) [7] tested sixteen different pose-image combinations and found that the supine-side-closed-mouth-base position model achieved the highest AUC (0.864), demonstrating strong sensitivity and specificity (~0.8).

Wang et al. (2023) [25] developed a MixMatch semi-supervised learning model using ResNet18 and achieved an AUC of 0.9435. Both sensitivity and specificity exceeded 89%, even with only 30% labeled data. Xia et al. (2024) [26] tested facial, traditional, and combined models. The facial model slightly outperformed the others (AUC 0.779), suggesting that bedside tests add marginal value.

In contrast, models based solely on clinical parameters performed less robustly; for example, those by Kim et al. (2021) [23] and Yamanaka et al. (2022) [27]. While the Balanced Random Forest model by Kim et al. [23] achieved an AUC of 0.79, others demonstrated moderate to poor predictive ability, thereby reinforcing the superiority of image-based models for this application.

The wide range of AUC values, from 0.6465 (Tavolara et al. [24]) to 0.9435 (Wang et al. [25]), reflects variability in methodology, datasets, and model architecture. However, consistent patterns emerged: models that integrated multiple angles, deep learning frameworks, and semi-supervised learning tended to outperform simpler or more traditional approaches.

The types of data input varied widely. Although all the original studies focused on image-based models, the diversity of facial views (frontal, lateral, and supine) and additional clinical parameters resulted in substantial heterogeneity. Hayasaka et al. were unique in that they employed supine imaging and demonstrated its superiority, particularly for patients who were unable to sit up.

The number of images per patient ranged from four (Kim et al., 2024 [22]) to 16 (Hayasaka et al. [7]), with studies such as Wang et al. [25] and Xia et al. [26] using 5–7 viewpoints. Despite these differences, most studies achieved performance comparable to or superior to that of traditional assessments, such as the Mallampati or LEMON scores. This reinforces the clinical value of AI-based approaches.

Notably, some models, such as those by Kim et al. (2021) [23] and Yamanaka et al. (2022) [27], relied solely on demographic and physiological data, including age, sex, neck circumference, and thyromental height. Image capture conditions, patient positioning, and anatomical variations significantly influence model performance; however, these factors are not uniformly described or controlled [30]. This raises concerns about reproducibility and generalizability, especially in settings that differ from the training environment [31].

Sample size was found to be a significant factor in determining the robustness of a model. Silvey and Liu (2024) [32] established minimum requirements for training sets to stabilize area under the curve (AUC) scores for AI algorithms. Using these benchmarks, only studies with more than 1000 image samples (e.g., Wang [25] and Kim [22]; Xia [26] and Hayasaka [7]) met the criteria for model stability. Tavolara’s smaller dataset (n = 505) likely limited the generalizability of their model.

Also, Xia et al. [26] addressed the class imbalance between difficult and non-difficult airway cases by randomly duplicating the minority class until parity was achieved. While this approach increases dataset balance, it may inadvertently increase the risk of overfitting by reducing variability within the difficult airway class. The lack of augmentation or synthetic data generation limits the model’s exposure to diverse anatomical presentations. These issues underscore the necessity of more robust imbalance-handling techniques, such as generative adversarial networks (GANs), focal loss functions, and data augmentation pipelines that preserve anatomical realism.

Another recurring limitation was ethnic homogeneity. Six of the original studies were conducted in East Asian populations (Japanese, Chinese, and Korean), with minimal representation from other ethnic groups. Since craniofacial morphology significantly influences airway anatomy, the performance of these models in ethnically diverse populations is uncertain. Although these models show promise with their high AUC values, validation in multi-ethnic cohorts remains essential.

A further methodological concern that applies to almost all of the included studies is the potential for publication and reporting bias. High-performing models are more likely to be submitted and accepted for publication than models with marginal or negative results. Due to the limited number of diverse studies and the absence of a quantitative meta-analysis, we could not formally evaluate publication bias using funnel plots or statistical tests. Nevertheless, the consistently high AUCs reported in the literature should be interpreted cautiously, as they may overestimate real-world performance.

Additionally, substantial selection bias exists in the patient populations used to train and validate these models. With the exception of a few emergency department cohorts, most datasets comprised adults undergoing elective surgery with a relatively low prevalence of truly difficult airways. Almost no studies incorporated patients with pathologies associated with challenging intubation, such as head and neck tumors, craniofacial anomalies, and airway malacia. Pediatric populations and out-of-hospital emergency scenarios were nearly absent. Consequently, current AI models have largely been evaluated in the “easiest” segment of airway management, so their performance is likely lower in settings where airway management is most hazardous and unpredictable.

### 4.3. Clinical Applicability and Interpretability

Several studies highlight the clinical potential of AI models designed for real-world integration. For instance, Kim et al. [22] used smartphone images on purpose and examined how clinician experience affects outcomes. Their research shows that AI assistance can reduce inter-operator variability and enhance decision-making in settings with limited resources or in emergencies. Similarly, Wang et al.’s semi-supervised model emphasizes reducing the burden of data annotation while preserving predictive accuracy [25].

However, turning these promising prototypes into routine clinical decision-support tools requires overcoming several practical barriers that go beyond image capture. First, it is critical to integrate the models into existing preoperative assessment pathways, anesthesia information systems, and electronic health records in a way that minimizes additional clicks, duplicate data entry, and delays [33,34]. While stand-alone smartphone applications may be attractive for proof-of-concept work, without seamless interoperability and institutional support, they are unlikely to be used consistently in busy operating rooms or emergency departments [35]. Second, relying on algorithmic predictions to guide airway management has medicolegal implications. It is unclear how responsibility should be shared between clinicians and vendors when AI suggestions conflict with clinical judgment or when an unexpected, difficult airway event occurs despite a “low-risk” prediction [33,36]. Third, the infrastructure and maintenance requirements, including secure image storage, periodic model recalibration, and performance drift monitoring, carry non-trivial costs that must be weighed against any accuracy improvements [37]. Finally, clinicians will need targeted training in how to use these tools and understand their limitations, interpret uncertainty, and avoid overreliance on seemingly precise outputs [38].

These issues echo broader capability- and function-oriented reviews of AI in “smart healthcare” that emphasize technical performance as a necessary but insufficient condition for successful deployment. Ultimately, AI-based airway assessment will need to be evaluated within comprehensive perioperative and emergency care pathways [39]. Ideally, this evaluation will be conducted through pragmatic trials and implementation studies that assess usability, alert fatigue, workflow disruption, and the effects on patient outcomes and resource utilization downstream.

The significant noticeable feature, the application of which can be a great help for healthcare providers who work with bedridden patients, is the AI model developed by Hayasaka et al. (2021) [7]. Their model, which showed the best accuracy in the supine-side position, allows the opportunity to assess a patient’s airway without causing them any pain or discomfort of moving or holding them up, especially in cases when it is advised against changing a patient’s position. In comparison to traditional methods, such as the Mallampati test, ULBT, or modified LEMON, which would require measuring parameters of mouth opening, this AI model does not require physicians to even touch or disturb a patient. But Kim et al. [22] conclude that Hayasaka et al.’s [7] use of such specific pictures requires more thorough research into image positions and their replication possibility in real-life scenarios. The majority of the remaining six original paper authors used frontal face images, which also demonstrated high precision results, with the MixMatch model by Wang et al. [25] showing the best performance. All these models are easy to use since the trained model only focuses on the images themselves, without the need to manually label, pinpoint, or set landmarks for facial features. Moreover, they all showed higher accuracy results than conventional methods of evaluation. The said advantages allow the use of AI models in all hospital wards for patients who are going to have surgery under general anesthesia. Kim et al. [23] and Yamanaka et al. [27] implemented the use of baseline characteristics and parameters that presented results similar to conventional methods’ results, which did not demonstrate the necessity of using only non-image data. Furthermore, AI models would decrease the number of intubation tries that could cause serious consequences, such as hemorrhage, especially in emergency departments and out-of-hospital situations when a healthcare provider with no or little clinical experience in the management of a difficult airway has to perform intubation [40]. Considering that the ready-to-use AI models require only patients’ pictures, they would be easy to use in daily practice when a person has a phone at hand and good enough lighting for the picture. Importantly, studies such as those by Yamanaka et al. [27] incorporated intubation outcomes, such as first-pass success, as endpoints. This approach could bridge the gap between algorithmic predictions and patient-centered outcomes.

However, the “black box” nature of many DL models poses a challenge to their clinical adoption [41]. Although heatmaps and Grad-CAM visualizations are sometimes used to highlight important facial features that influence predictions (as in studies by Xia et al. [26] and Hayasaka et al. [7]), true interpretability remains limited. For clinicians to trust and utilize AI outputs, there must be greater transparency in model architecture and feature attribution [42]. Efforts should focus on explainable AI (XAI) frameworks that align predictive outputs with clinically understandable features [43].

Additionally, the ethical implications of deploying facial image-based models must be considered. Patient consent, data privacy, the potential misuse of biometric data, and algorithmic bias must be carefully considered before clinical deployment. While all of the included studies were retrospective and de-identified, future prospective studies and real-time applications will require stringent governance protocols.

### 4.4. Ethical and Regulatory Considerations

As AI becomes more integrated into clinical workflows, ethical considerations become paramount [44]. Using facial data introduces unique privacy and discrimination risks. Even when anonymization techniques are used, facial images are still identifiable [45]. Researchers must adhere to robust ethical standards, including obtaining informed consent for image use, implementing strict data security measures, and being transparent about the use of models.

Beyond privacy concerns, ensuring algorithmic fairness is a central ethical challenge for facial image-based airway models. None of the included studies reported pre-specified fairness metrics, subgroup performance analyses (e.g., by sex, ethnicity, or age), or bias-mitigation strategies [38]. The concentration of training data in East Asian populations is particularly concerning, given the known discrepancies in facial recognition systems across demographic groups. Without explicit fairness testing, these models may systematically underperform in underrepresented populations, thereby exacerbating existing disparities in perioperative risk. Future studies should, at a minimum, report stratified performance and adopt fairness metrics appropriate to the clinical context (e.g., equal opportunity or predictive parity) [46]. They should also transparently describe any bias-mitigation techniques used during model development. Regulatory frameworks and institutional governance should require such reporting before clinical deployment and mandate periodic audits as the case mix evolves over time [47].

Algorithmic bias is another critical concern. Since most models have been trained on homogeneous populations, there is a substantial risk that they may underperform in underrepresented groups, thereby exacerbating health disparities [48]. Therefore, regulatory frameworks must mandate demographic reporting, fairness audits, and bias mitigation strategies during model development.

From a legal standpoint, liability for AI-driven clinical decisions remains ambiguous. Clear guidelines must be established regarding clinicians’ responsibilities when using AI as a decision-support tool [44]. Furthermore, as AI tools enter commercial markets, regulatory oversight (e.g., by the FDA or EMA) will need to adapt to ensure safety, efficacy, and accountability.

### 4.5. Clinical Utility and Cost-Effectiveness

The performance gains observed in AI-based airway models must be interpreted in terms of clinical utility. While most of the included studies reported traditional discrimination metrics, such as the AUC, sensitivity, and specificity, none defined a minimal clinically important difference (MCID) in these measures that would justify their adoption over standard bedside tests. For example, an increase in the AUC from 0.75 to 0.85 might not result in fewer hypoxic events or unplanned surgical airway procedures if the prevalence of difficult airways is low or if the management strategy remains unchanged [49]. Additionally, none of the studies evaluated calibration, decision curve analysis, or net benefit at clinically relevant decision thresholds. Furthermore, none of the algorithms linked their predictions to hard patient outcomes in prospective trials.

Furthermore, no formal cost-effectiveness analyses were reported. Implementing AI-driven airway assessment will incur costs related to imaging hardware, software licenses, data storage, staff training, and ongoing model maintenance [50]. Potential benefits, such as a reduced incidence of failed intubation, fewer ICU admissions, and shorter operating room delays, remain speculative at this stage [51,52]. Therefore, future research should incorporate health economic evaluations and decision analytic modeling to determine whether the observed improvements in diagnostic accuracy justify the financial and organizational investment required for large-scale deployment [53].

### 4.6. Toward Standardization and Benchmarking

This review reveals the urgent need for standardization in the development and evaluation of AI models for airway prediction [54]. First, there must be a consensus on which metrics, beyond AUC, should be universally reported. Second, the datasets used for training and testing models should be publicly accessible or clearly documented in terms of demographic composition, image acquisition protocols, and annotation methods.

Standardized image acquisition protocols (e.g., patient position, lighting, and device resolution) would greatly enhance cross-study comparability. Benchmark datasets, similar to ImageNet or MIMIC-III in other fields, are currently lacking in airway management. Developing such a dataset under the auspices of international anesthesiology societies could serve as a reference for model validation and benchmarking.

Moreover, validation across real-world settings is critical. Most of the models reviewed were trained using data collected in elective surgical contexts. Their performance in emergency intubations, pediatric populations, or resource-limited settings is unknown. Future studies must incorporate these scenarios to establish external validity.

### 4.7. Future Directions

The use of AI models can get a lot easier if they are developed as applications, as previously mentioned by the authors. Therefore, future research needs to focus on what image angles an application should use for the assessment, as well as how the template or borders for pictures should be presented, to also make the user experience easier when working with the app interface for the first time [10]. The preparation of thorough and clear instructions for first-time users of an application would be a great help to healthcare providers.

In both cases, if AI models are used as a phone application or a website, the important factor that should be considered is the clear and easily understandable presentation of the final decision by the model regarding the airways assessment. That is why the predictive parameters and their reporting values should be given in a certain universal template for all models. Having a standardized, universally accepted deployment template for AI-based airway assessment tools would ensure a consistent user experience and interpretation across different platforms [37]. In addition to harmonizing the clinical workflow, these systems could incorporate measures of typicality, quantifying how similar a given patient is to the training population of the model. These applications could flag out-of-distribution cases and alert clinicians when predictions may be less reliable, supporting more cautious interpretation. This approach would be especially valuable in addressing ethnic, anatomical, and demographic variability. It would provide transparency about the generalizability of models in diverse patient populations.

The difference in the presentation of results and the need to spend additional time interpreting the data could significantly delay the next procedures. In case of emergencies, every second counts; thus, the potential malfunction should be addressed in advance.

One promising approach is to use deep learning to automatically extract facial landmarks, such as jaw angle, interocular distance, and chin-throat curvature, and analyze the geometric relationships among them [55]. These derived anatomical features could then serve as inputs for simpler, more interpretable models, such as decision trees or rule-based systems. This would bridge the gap between complex DL pipelines and clinician-friendly outputs.

Pediatric and emergency airway scenarios were underrepresented in the current literature [56]. Given the anatomical and clinical differences in these populations, future studies should validate AI tools specifically in these high-stakes settings

The further study should also concentrate on the matter of applying the discussed AI models on a larger scale to cover data from several hospitals or even a city’s hospitals. This would increase accuracy over time due to the fact that the AI model would get to train after each case is added to the database. Additionally, AI models should be trained to cover databases of patients of different nationalities to avoid bias and learn to differentiate ethnicities and make subcategories of feature specifications for more precise judgment.

### 4.8. Limitations

There were several limitations of this research project identified. First, although the review followed the PRISMA 2020 guidelines, the protocol was not registered in PROSPERO or a similar repository. The absence of prospective registration introduces the possibility of post hoc refinement of the inclusion criteria or analytical focus. We attempted to mitigate this risk by defining the protocol in advance and providing the key elements in the Supplementary Materials. However, some residual risk of selective reporting remains.

Second, the ten articles included for this study could be considered not enough for a comprehensive analysis of artificial intelligence models in the field of difficult airways assessment and their application in clinical practice. Third, the study mainly focused on neural networks, machine or deep learning models that used only facial images as a primary training data source. Therefore, models that relied on medical images, such as CT, MRI, or ultrasound scans, were not assessed for their application in difficult airways management.

We could not formally assess publication bias or small-study effects due to the limited number of included studies and their heterogeneity, as well as the absence of a pooled meta-analysis. It is still possible that underperforming or negative AI models are underreported, which could make published systems appear more superior than they actually are. Fourth, most of the included studies were conducted at single centers and focused almost exclusively on East Asian populations and adult patients undergoing elective surgery. Pediatric patients, emergency intubations, and individuals with structural airway pathology were rarely represented, if at all. This restricted case mix means that our synthesis may overestimate performance in high-risk, real-world scenarios, where difficult airways are more frequent and consequential from a clinical standpoint.

Finally, we did not conduct a comprehensive search of IEEE Xplore, the ACM Digital Library, or ArXiv. Although this approach may have resulted in the omission of some technically relevant AI models, preliminary research indicated that these repositories rarely contained studies with clinical evaluations and diagnostic accuracy data that aligned with our inclusion criteria.

## 5. Conclusions

Taking into consideration all the results discussed above, we can indeed conclude that our hypothesis was supported: artificial intelligence models that use facial recognition to assess image datasets even outperform bedside assessment tests and visual diagnostic techniques. The current best AI model among those developed by the original paper’s researchers is the MixMatch model with 30% labeled data, due to its high evaluation accuracy. However, there were limitations of this study, such as having 10 articles used for this analysis, and the heterogeneity of the data used by the various models makes it difficult to draw clear conclusions. Moreover, further research should be conducted in the field of application development to achieve high accuracy in difficult airways identification and to provide an easy and accessible interface for app users across all medical specialties and clinical experience levels. These models can also be improved with the addition of medical images for better assessment of certain anatomical features of patients, based on which a more accurate assessment of difficult airways could be conducted.

## Figures and Tables

**Table 1:** General classification of featured papers.

Authors	Article Type	Research Purpose	Evaluation Methods ^a^	AI Models ^a^
Chen et al. (2024) [10]	Review	To describe current AI applications in difficult airway assessment; To determine the advantages and disadvantages of traditional and high-end methods.	No specific measurements indicated	Machine learning (algorithms: random forest, facial recognition); Deep learning (CNN ^b^, RNN ^b^) Computer vision.
De Rosa et al. (2025) [17]	Review	To investigate the effect of AI models that use photographs and clinical assessment for managing difficult airways.	Fully automated model of predicting difficult endotracheal intubation with 900 patients’ face images: AUC 0.81 DL prediction model with face images: AUC 0.7105, high sensitivity, low specificity. DL and CNN model using facial images for difficult airway classification: AUC 0.864, sensitivity 81.8%, specificity 83.8%, accuracy 80.5% Gradient Boosting clinical assessment predictive model made with 10 DL and machine learning algorithms: AUC > 0.8, accuracy > 90%, precision 100%.	DL (deep learning) segmentation of MRI, CT, X-ray images: CNN, for biomedical image (U-Net), Mask region-based CNN (Mask R-CNN). Facial recognition; Machine learning.
Hayasaka et al. (2021) [7]	Original	To create a deep learning model based on CNN for the detection of intubation difficulty using facial images of patients.	Supine-side-closed mouth-base position model: Accuracy 80.5%, sensitivity 81.8%, specificity 83.3%, AUC 0.864. Supine-side-opened mouth-base position model: AUC 0.758. Supine-side-closed mouth-backbend position model: AUC 0.727. Sitting-front-opened mouth-base position model: AUC 0.592. Sitting-side-opened mouth-base position model: AUC 0.387.	16 positions AI models developed by mixing and combining the following options (see Figure S1, Supplemental Materials): Supine-Front or Supine-Side OR Sitting-Front or Siting-Side; Closed mouth or Opened mouth; Base or Backbend position.
Kim et al. (2024) [22]	Original	To create a practical artificial intelligence model that would use patient bedside images taken with a smartphone.	Deep learning model for predicting difficult direct laryngoscopy (DDL): ROC AUC 0.81–0.88, recall/sensitivity 0.63–0.9, precision 0.7–0.88, F1-score 0.72–0.81.	EfficientNet-B5 deep learning model (pre-trained model derived from ImageNet database).
Kim et al. (2021) [23]	Original	To develop a model for difficult laryngoscopy prediction using neck circumference and thyromental height.	Machine learning model using BRF algorithm (best): AUROC 0.79, AUPRC 0.32, sensitivity 0.90, specificity 0.58, accuracy 60%. Machine learning model using MLP algorithm (worst): AUROC 0.63, AUPRC 0.17, sensitivity 0.49, specificity 0.60, accuracy 61%. Machine learning model using the LR algorithm (worst): AUROC 0.63, AUPRC 0.18, sensitivity 0.66, specificity 0.56, accuracy 57%.	Machine learning: Balanced Random Forest (BRF); Extreme Gradient (XG-) Boosting (XGB); Light Gradient Boosting Machine (LGBM); Multi-layer Perceptron (MLP); Logistic Regression (LR).
Matava et al. (2020) [15]	Review	To present artificial intelligence applications’ potential in pediatric anesthesiology; To identify limitations of novice methods and areas for further research.	Face analysis model: 77.9% PPV ^c^. Machine vision model for detection and labeling of vocal cords and tracheal anatomy: Sensitivity 0.87, specificity 0.89. Glottis locating machine learning algorithm: Accurate prediction 74.5%, adjacent prediction 21.5%.	Machine learning (also random forest algorithm); Machine vision (real-time vocal cords classification and labeling). Machine learning algorithm for identifying glottis location from laryngeal images.
Tavolara et al. (2021) [24]	Original	To develop a deep multiple-instance learning model for the detection of difficult-to-intubate patients; To prove the better performance of the authors’ new method compared to conventional tests.	First strategy—retrain the last layer of FRFE: Inner corner of eyes and bottom center of lip model’s ensemble performance: Sensitivity 69.74%, specificity 64.47%, AUC 0.6465. No alignment, for neck features (the same performance for ensemble): Sensitivity 68.42, specificity 61.84. Second strategy—FRFE with MIL ^d^: Ensemble model’s performance: Sensitivity 73.68, specificity 68.42, AUC 0.7105. Cross-validation results: For the FRFE model: Inner eye and bottom lip: sensitivity 0.8158, specificity 0.4868. Outer eye and bottom nose: sensitivity 0.8143, specificity 0.3286. No alignment: sensitivity 0.8026, specificity 0.5263. For the MIL model: Sensitivity 0.8158, specificity 0.5263.	33 CNNs (3 face alignments, 11 face regions) training model built to make the base of Face Region Feature Extractor (FRFE) for patient face feature extraction.
Wang et al. (2023) [25]	Original	To create a semi-supervised deep learning model that will focus on both intubation and mask ventilation difficulties; To overcome challenges of existing AI-based models and methods.	MixMatch with ResNet18 as backbone network (30% data labeled) results: Accuracy 90.00%, sensitivity 89.58%, specificity 90.13%, F1 81.13%, AUC 0.9435. Upper Bound (100% data labeled) results: Accuracy 90.50%, sensitivity 91.67%, specificity 90.13%, F1 82.25%, AUC 0.9457.	Semi-supervised deep learning models (SSL): MixMatch, Π-Model, Mean Teacher, visual adversarial training (VAT), Pseudo-Label. Backbone networks: ResNet18.
Xia et al. (2024) [26]	Original	To invent an AI model for classification of videolaryngoscopy cases as difficult and non-difficult based on views; To research this AI model’s feasibility on a large scale.	Three image positions (upper lip bite, mouth open, tongue extension): AUROC > 0.7. Facial model: Sensitivity 0.757, specificity 0.721, AUROC 0.779. Combined model: Sensitivity 0.767, specificity 0.729, AUROC 0.778. Facial model and combined model: *p* = 0.907. Traditional model: Sensitivity 0.738, specificity 0.687, AUROC 0.754. Facial model and traditional model: *p* = 0.343.	Computer vision: facial analysis; Deep learning backbone network: ResNet18 (18-layer model); Combined model (logistic regression) includes eight variables: MP ^e^; mandibular protrusion; TMD ^e^; inter-incisor gap; positions: head up, lateral, mouth open, tongue extended. Facial model (LightGBM ^f^) includes four image positions: head-up, mouth open, lateral, tongue extension; Traditional model (logistic regression) based on non-image data.
Yamanaka et al. (2022) [27]	Original	To use machine learning for difficult airways and first-pass success prediction.	Ensemble model for difficult airway prediction: C-statistics 0.74, sensitivity 0.67, specificity 0.70, PPV 0.09, NPV 0.98. Ensemble model for first-pass success prediction: C-statistics 0.81, sensitivity 0.79, specificity 0.67, PPV 0.85, NPV 0.57.	Machine learning models (for each outcome prediction): Logistic regression; Random forest; Gradient boosting decision tree; Multilayer perceptron; K-point nearest neighbor; XGBoost; Ensemble model.

^a^ For review articles, indicated evaluation methods and AI models were the most discussed in the paper. ^b^ CNN, convolutional neural network; RNN, recurrent neural network. ^c^ PPV, positive predictive value; NPV, negative predictive value. ^d^ MIL, multiple instance learning. ^e^ MP, Mallampati test; TMD, thyromental distance. ^f^ LightGBM, Light Gradient Boosting Machine.

**Table 2:** Comparison of review papers.

Criteria	Chen et al. (2024) [10]	De Rosa et al. (2025) [17]	Matava et al. (2020) [15]
Number of publications investigated, n	Not clearly indicated; References: 67.	847 (titles and abstracts reviewed), 31 of them (used for full review).	Not clearly indicated; References: 27.
Data type	Facial images; Cervical spine lateral X-ray images.	Facial images (from different views, from 4–16); Medical images (X-ray, CT, MRI).	Face images and videos; pediatric bronchoscopies; laryngeal images.
Comparison with existing methods	Bedside assessment (Mallampati test, TMD ^a^, ULBT ^a^). Disadvantages: set cutoff values, subcategorized results, subjective. Comprehensive tests (LEMON): Disadvantages: complex, time-consuming. X-ray: Advantages: Clear visual of skeletal structures. CT, MRI: Advantages: Detailed view, visible anatomical structures. X-ray, CT, MRI: Disadvantages: radiation, time-consuming, expensive. Ultrasound: Advantages: Laryngoscopy-visible anatomical structures (tongue, epiglottis, glottis), and not (hyoid bone, cricoid cartilage, soft tissues of neck); low cost; availability. Computer-aided airway reconstruction and three-dimensional (3D) printing techniques: Advantages: Opportunity for forming the safest plan for operation, encouraging new intubation device production. Disadvantages: high cost, limited availability.	CT scan segmentation for airway diameter, wall thickness: Manual: >15 h for each scan; Semi-automated: >2.5 h for each scan; Automated: not indicated; Advantage: precision, no bias for the operator. Disadvantages of these methods: time-consuming, image feature dependence.	No existing traditional or conventional methods discussed.
Conclusions	AI algorithms involving face images are recommended because they can: increase sensitivity and specificity of diagnosis; be a reliable guide for new specialists. In studies, deep learning models are mostly described in a “black box” manner, which significantly decreases the model’s interpretability.	Research about the application of AI in MRI is limited. Most developed models were created in limited conditions, such as using data of patients scheduled for surgery. This aspect lowers the predictability of results if applied to unexpected or emergency cases. There may be limitations of studies to certain ethnicities, decreasing the possibility of applying the model worldwide.	Machine learning models could be developed to serve as secondary and supplementary tools of a patient’s diagnosis, making the physician’s clinical decision and assessment the main judgment of the case.
Future perspectives	Difficult airway assessment apps: The development of apps using face images of patients has the potential to improve difficult airway management. Nowadays, the operation is still time-consuming and complex due to the selection of identification points by yourself, an issue that could be fixed and simplified in the future.	AI in MRI: An image recognition tool can help in identifying neck or airway obstructions (high-arched palate, narrow oropharynx, short neck) causing difficult airways from MRI scans.	GPS guide: Machine learning algorithms can be developed in the direction of GPS guides for the procedures of video laryngoscopy and bronchoscopy, which would greatly assist physicians and residents still new to managing difficult airways in children.

^a^ TMD, thyromental distance. ULBT, upper lip bite test.

**Table 3:** Comparison of original research papers in terms of accessibility of methods, conclusions, and future perspectives.

Authors	Accessibility of Methods (for Reproducing the Models)	Conclusions	Future Perspectives
Hayasaka et al. (2021) [7]	The authors used the already existing VGG16 model and its modified version for training and developing the CNN model. The learning rates, epochs, and data processing steps were also included. The procedure did not have complex algorithms.	The best predictive model was found to be the one using the supine-side-closed mouth-base position. Photos taken in a sitting position could not effectively be assessed for difficult intubation. But, there were limitations: Patients selected for this study were scheduled for surgery, which might decrease the applicability of the same results in emergency cases. The study may not be applicable in pediatric care, as it was designed based on adult patients. The area of research was limited due to being conducted in one hospital, and the reluctance of older people to take images for data collection.	The authors discussed the opportunity of developing an application. In addition, they believe it would be possible to create a model that can work with huge amounts of data, including a higher number of face images in a larger area of study coverage.
Kim et al. (2024) [22]	The details of data preprocessing are presented with equations and supporting schemes. Additionally, authors included the specifics for each fold for cross-validation.	The model showed good performance with a focus on practical application in clinical situations, using simple and limited data. Limitations of other studies were considered and resolved for this study: Hayasaka et al. (2021) [7] used tightly controlled images that would not be possible to achieve in every situation, so they developed more flexible imaging setups in their model, used fewer photos, and a simple smartphone camera. Tavolara et al. (2021) [24] used a model that learns in two steps, which was not a very optimized way. Thus, the authors used an end-to-end framework of deep learning. But there were still limitations: Number of image views limited to four; Videolaryngoscopy use was not considered, Certain variability in CL classification due to different levels of physicians’ training, clinical experience amount, and subjectivity. Low incidence of DL in real life, possibly affecting the model accuracy due to high incidence in training.	Further research should focus on improving the classification of laryngeal views to avoid issues of overreporting CL grades 3–4 cases or misclassifying cases. Furthermore, future studies have to rely on more anesthesiologists to objectively evaluate the incidence rate of DL compared to real-life statistics, which would still be enough for the model’s training.
Kim et al. (2021) [23]	Authors included details about each of the machine learning algorithm packages, a program to run the mentioned algorithms, and equations for the calculation of certain parameters. Thus, it can be considered an appropriate amount of data for model reproduction.	The overall performance of the models was good but needs the addition of new predictors and training based on this data. However, ensemble models presented close to or higher than other references’ results. There were limitations as follows: No model showed very high AUROC or AUPRC, the latter having low results. No model excelled in two or more evaluation measures. The model training and validation data can be challenging to use on other ethnicities or children due to the main audience of the study being adults, mostly Korean.	More data (predictors, variables) are needed for the improvement of the model’s performance as a predictive tool. The way to overcome the weaknesses of each model would be to apply an ensemble model with both high sensitivity and specificity.
Tavolara et al. (2021) [24]	The procedures, both of data preprocessing and processing, are described in detail. It includes the schematic illustrations, descriptions, learning rates, and epoch numbers for FRFE model recreation.	The model presents a huge improvement in the field of AI detection of difficult airways, focusing on the facial features of patients. This model showcases high sensitivity and specificity compared to bedside tests, significantly outperforming them. However, there were limitations: Face alignment did not align all patients’ images on the same scale. Face alignment should be focused on individual features, not the whole face. CASIA-Webface did not pass augmentation, like the patients’ dataset.	The model can be further developed using not only frontal face images, but also profile pictures. Profile angle would help with the analysis of jaw and neck features. Additionally, face ratios between landmarks could be useful in predicting difficult airways by comparing different ratios.
Wang et al. (2023) [25]	The authors provided a detailed description of algorithms (all four parts of MixMatch SSL) and comparison results for identifying the best backbone network to work with. They included information about programs they run experiments in, epochs, and learning rates. Also, data preprocessing and extraction steps are given.	SSL models with 30% labeled data show results of high precision and accuracy, close to those of anesthesiologists with more than 5 years of experience, proving that providing 100% labeled data is not necessary. This solution solves the issue of time-consuming manual labeling of images. Multi-channel fusion of numerous images from different angles and key difficult airway indicators increases the reliability of the method.	The SSL model based on MixMatch can be improved by using data from several hospitals across China, which would significantly decrease single-center and possible bias factors’ impact. The model also has the potential to be developed as an app for easy access anywhere and anytime.
Xia et al. (2024) [26]	Authors included a description of algorithms for model development along with supplementary information on procedures for initial processing of data and application to the model. Name of the program, its settings, epochs, learning rates, and parameters were presented in the paper.	There was a significant difference in baseline characteristics between groups of difficult and non-difficult videolaryngoscopy patients. Combined model (images and clinical assessment) and facial model (only including image analysis) performed with no significant difference, proving no need for clinical examination in assessing for difficult videolaryngoscopy. The AI model showed better results compared to traditional and a no-image data-based model. Heatmaps helped in the identification of important facial features for difficult airway assessment, presenting them in red and yellow.	The methodology of this research using both ResNet18 and LASSO makes it possible to use general images, without the need for a large number of images for the assessment. Further development for a larger scale can allow application of this model in multi-center studies, including Caucasian populations, as well as Asian.
Yamanaka et al. (2022) [27]	Although authors do not include links or package details about each of the machine learning algorithms, since the said articles are common to find on the internet, and program names are included, the data should be enough to understand and reproduce the model.	The predictive model based on machine learning that uses predictors collected in routine observation performed better than conventional methods. The limitations highlighted by the authors include the following: Possibility of bias in data reporting and measurement; Absence of data on the professional competency of intubators since the measurement is difficult to conduct; Possible challenges in the interpretability of models by machine learning; Non-adaptability of the model to children’s diagnosis; Limitation of the study to the Japanese population.	The development and integration of machine learning for the prediction of intubation outcomes can help to improve the airway management practice and conditions of ill patients in emergency departments.

## Data Availability

No new data were created or analyzed in this study. Data sharing is not applicable to this article.

## Supplementary Materials

The following supporting information can be downloaded at: https://www.mdpi.com/article/10.3390/jcm14238600/s1, Figure S1: AI models based on 16 positions of patient images; Figure S2: Heat maps of supine-side-closed mouth-base position; Figure S3: Average heat map images of easy and difficult airway cases; Figure S4: Algorithm illustration of CASIA-Webface based training. (Image of Muhammad Ali, from Wikimedia Commons, public domain); Figure S5: Multiple instance learning (MIL) model with application of face region feature extractor (FRFE); Figure S6: Examples of patients images taken from 9 different views; Figure S7: Illustration of seven image positions; Figure S8: Heat maps using gradient-weighted class activation maps (Grad-CAM++); Figure S9: Patient photographs from four views for model training; Figure S10: Gradient-weighted class activation mapping pictures (Grad-CAM). Table S1: Database-specific search strategies for identifying AI/ML models for difficult airway prediction (search period: 1 January 2020–July 2025); Table S2: Core protocol for the systematic review of AI/ML models for predicting difficult airways; Table S3A: Comparison of original research papers in terms of participants, data and existing methods; Table S3B: Comparison of original research papers in terms of participants, data and existing methods.

## Abbreviations

The following abbreviations are used in this manuscript:

AI	Artificial Intelligence
ML	Machine Learning
DL	Deep Learning
CNN	Convolutional Neural Network
RNN	Recurrent Neural Network
MIL	Multiple Instance Learning
FRFE	Face Region Feature Extractor
SSL	Semi-Supervised Learning
GAN	Generative Adversarial Network
XAI	Explainable Artificial Intelligence
CDSS	Clinical Decision Support System
AUC	Area Under the Curve
AUROC	Area Under the Receiver Operating Characteristic Curve
AUPRC	Area Under the Precision–Recall Curve
ROC	Receiver Operating Characteristic
PPV	Positive Predictive Value
NPV	Negative Predictive Value
PLR	Positive Likelihood Ratio
NLR	Negative Likelihood Ratio
TMD	Thyromental Distance
ULBT	Upper Lip Bite Test
LEMON	Look, Evaluate, Mallampati, Obstruction, Neck mobility (airway assessment system)
ASA PS	American Society of Anesthesiologists Physical Status
CL	Cormack–Lehane (classification)
ECG	Electrocardiogram
NBP	Non-invasive Blood Pressure
SpO_2_	Peripheral Oxygen Saturation
EtCO_2_	End-tidal Carbon Dioxide Concentration
BRF	Balanced Random Forest
LGBM	Light Gradient Boosting Machine
XGB	Extreme Gradient Boosting
LR	Logistic Regression
MLP	Multi-Layer Perceptron
ResNet18	Residual Neural Network with 18 layers
VGG16	Visual Geometry Group 16-layer Network
Grad-CAM	Gradient-Weighted Class Activation Mapping
BMI	Body Mass Index
MRI	Magnetic Resonance Imaging
CT	Computed Tomography
AU	Arbitrary Unit
IQR	Interquartile Range
NDDL	Non-Difficult Direct Laryngoscopy
DDL	Difficult Direct Laryngoscopy
NDL	Non-Difficult Laryngoscopy
DL (clinical)	Difficult Laryngoscopy
NC	Neck Circumference
TMHT	Thyromental Height
QUADAS-2	Quality Assessment of Diagnostic Accuracy Studies-2
FDA	U.S. Food and Drug Administration
EMA	European Medicines Agency
MIMIC-III	Medical Information Mart for Intensive Care III
GANs	Generative Adversarial Networks
