# Supplementary material for: Artificial Intelligence for Predicting Difficult Airways: A Review
Source: J Clin Med. 2025 Dec 4;14(23):8600. doi: 10.3390/jcm14238600 (PMC12693205; doi:10.3390/jcm14238600)
Supplement: Supplementary file 1 [file jcm-14-08600-s001.zip › jcm-4003083-supplementary.pdf]

Supplemental Figures

Disclaimer. All supplementary figures are available online. No modifications were made to the original published figures. All figures were gathered into a single supplemental file solely to enhance transparency and reproducibility of the studies cited in this review. Each figure originates from previously published, open-access sources under the Creative Commons Attribution (CC BY 4.0) license. Proper citations and credit lines are included in the corresponding figure legends.

Figures from S1 to S3 illustrate the main process steps of the research by Hayasaka et al. (2021) [7].

Figure S1 shows 16 positions of how patient photographs were taken.

Figure S1. *AI models based on 16 positions of patient images.*

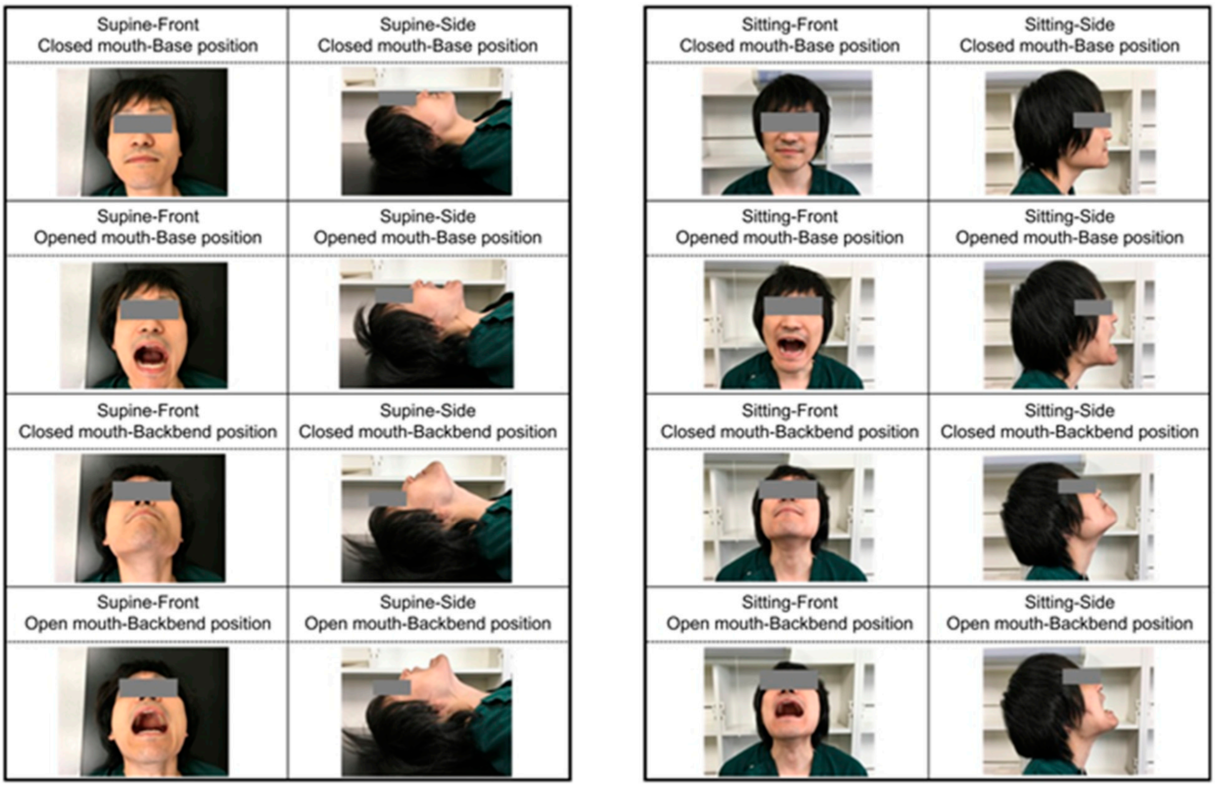

Note. Image positions were set by altering sitting or supine position, opening or closing mouth, holding neck in still, base or backbend position. (Hayasaka et al., 2021) [7].

© Hayasaka, T., Kawano, K., Kurihara, K. et al. Creation of an artificial intelligence model for intubation difficulty classification by deep learning (convolutional neural network) using face images: an observational study. j intensive care 9, 38 (2021). https://doi.org/10.1186/s40560-021-00551-x. (CC BY 4.0) https://creativecommons.org/licenses/by/4.0/

Figures S2 and S3 are the heat maps of patients' images from different positions. Yellow and red areas are determined to be important for the assessment of difficult airways and making a decision about intubation class (easy or difficult). The difference between RGB images of easy and difficult to intubate cases can be seen in Figure S3.

Figure S2. *Heat maps of supine-side-closed mouth-base position.*

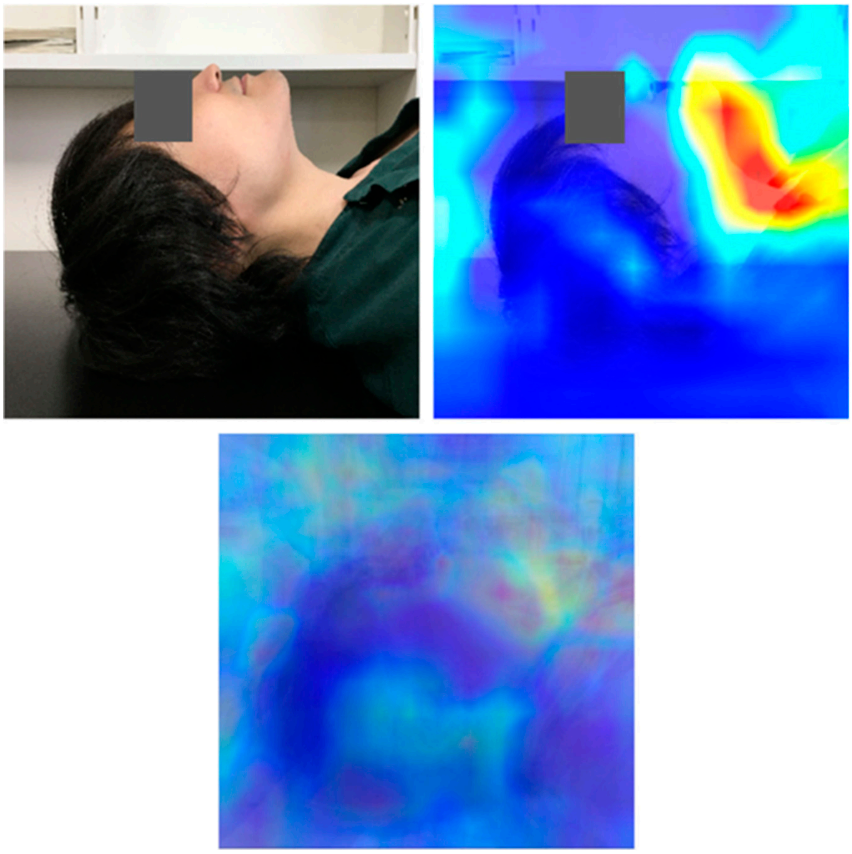

Note. The pictures are heatmaps of supine-side-closed mouth-base position of a patient: yellow and red areas determined to be important for difficult intubation detection. (Hayasaka et al., 2021) [7].

© Hayasaka, T., Kawano, K., Kurihara, K. et al. Creation of an artificial intelligence model for intubation difficulty classification by deep learning (convolutional neural network) using face images: an observational study. j intensive care 9, 38 (2021). https://doi.org/10.1186/s40560-021-00551-x. (CC BY 4.0) https://creativecommons.org/licenses/by/4.0/

Figure S3. *Average heat map images of easy and difficult airway cases.*

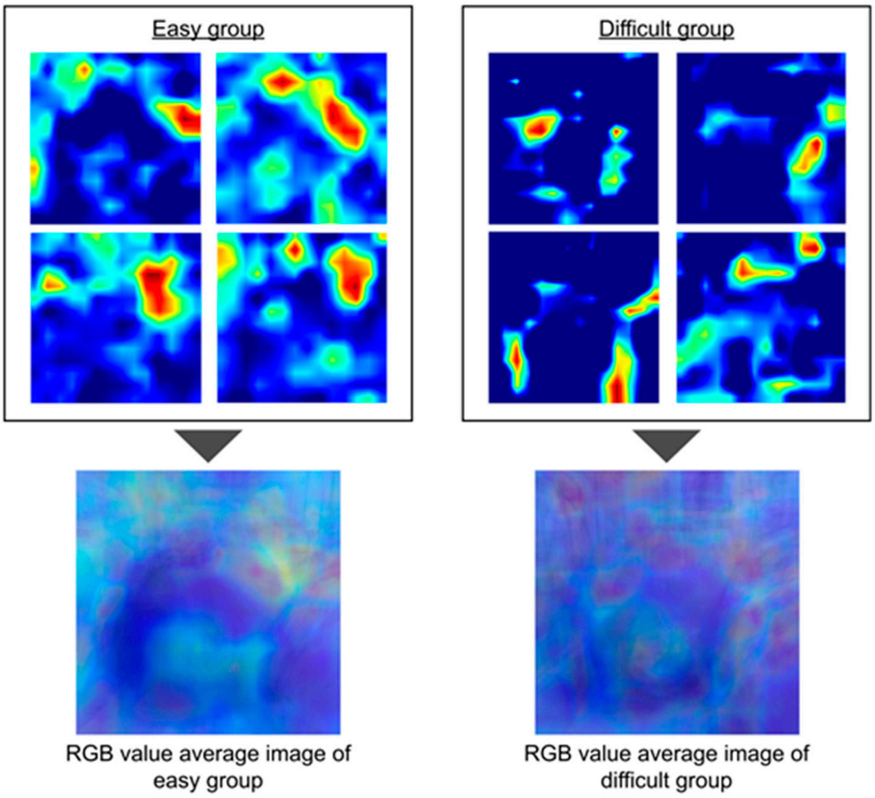

Note. Heatmaps pictures of supine-side-closed mouth-base position of easy (left top corner) and difficult (right top corner) intubation cases. The bottom two pictures are average RGB images of two classifications (Hayasaka et al., 2021) [7].

© Hayasaka, T., Kawano, K., Kurihara, K. et al. Creation of an artificial intelligence model for intubation difficulty classification by deep learning (convolutional neural network) using face images: an observational study. j intensive care 9, 38 (2021). https://doi.org/10.1186/s40560-021-00551-x. (CC BY 4.0) https://creativecommons.org/licenses/by/4.0/

Figures S4 and S5 show the main processes completed by Tavolara et al. [20] (2021) during the training of convolutional neural network models for creation of Face Region Feature Extractor (FRFE). Figure S5 demonstrates in the detail the methodology of multiple instance learning application to FRFE.

Figure S4. *Algorithm illustration of CASIA-Webface based training. (*Image of Muhammad Ali, from Wikimedia Commons, public domain.)

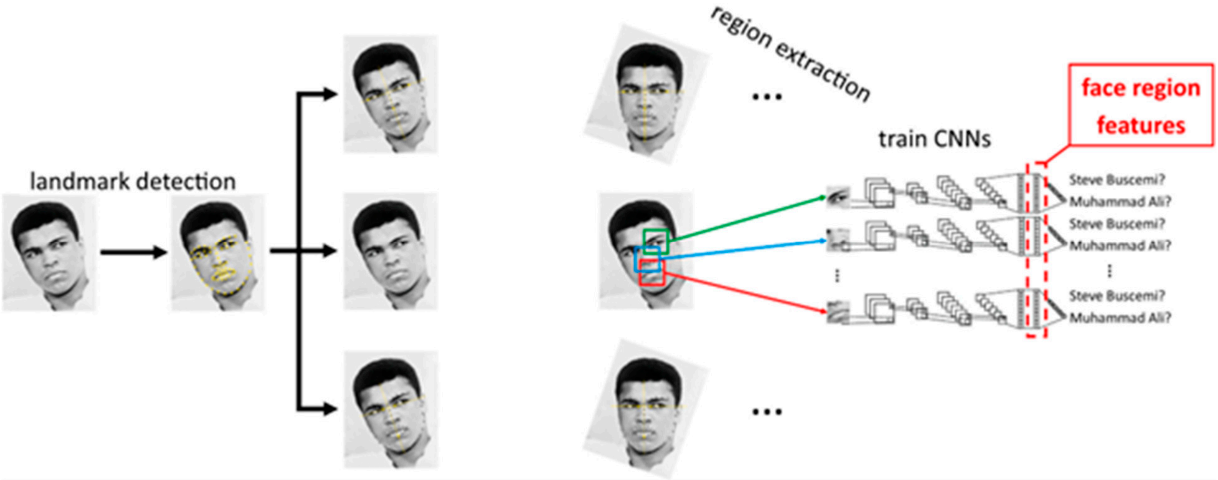

Note. The diagram of landmark detection, face alignment, face region extraction and CNN training procedures based on CASIA-Webface. (Tavolara et al., 2021) [24].

© Tavolara, T.E.; Gurcan, M.N.; Segal, S.; Niazi, M.K.K. Identification of difficult to intubate patients from frontal face images using an ensemble of deep learning models. Comput Biol Med 2021, 136, 104737, doi:10.1016/j.compbiomed.2021.104737 (CC BY-NC-ND 4.0) https://creativecommons.org/licenses/by/4.0/

Figure S5. *Multiple instance learning (MIL) model with application of face region feature extractor (FRFE).*

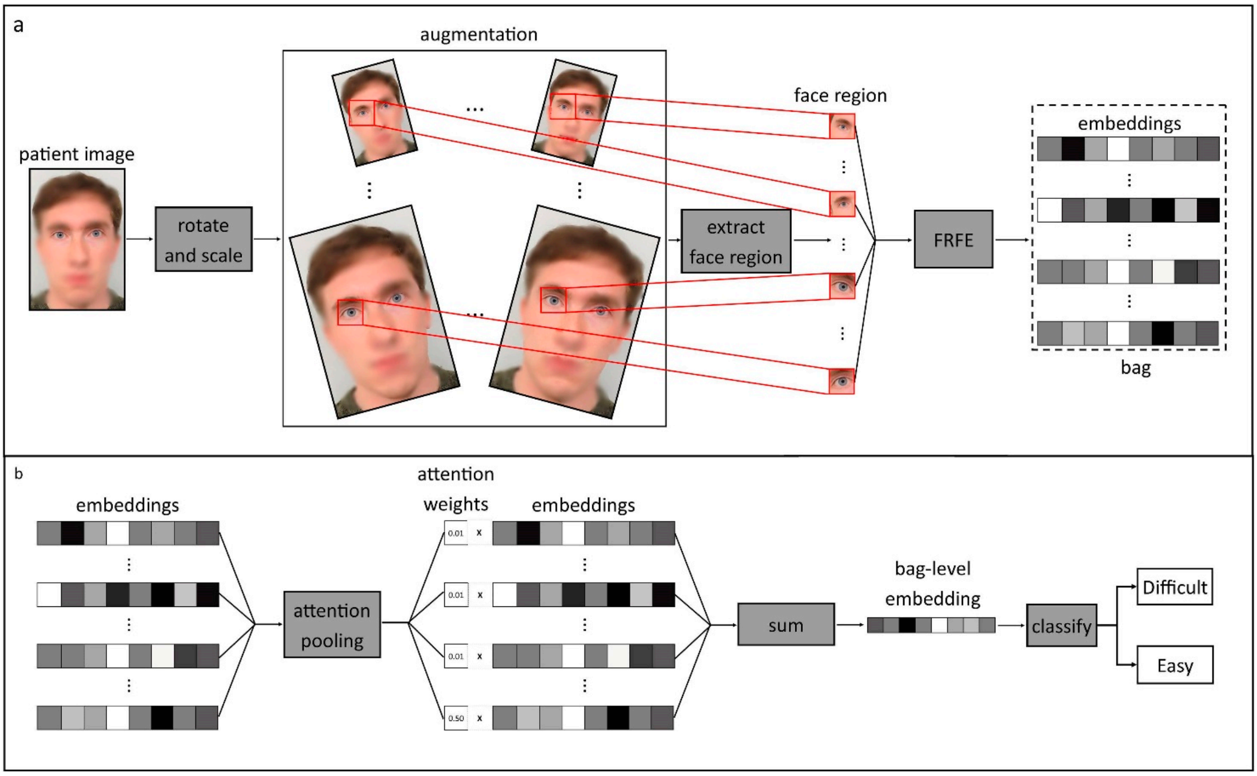

Note. The demonstration of the process of MIL model processing patient images (augmentation, face feature extraction, collection labeling, classification) ("Patient image" is of first author). (Tavolara et al., 2021) [24].

Reproduced from © Tavolara, T.E.; Gurcan, M.N.; Segal, S.; Niazi, M.K.K. Identification of difficult to intubate patients from frontal face images using an ensemble of deep learning models. Comput Biol Med 2021, 136, 104737, doi:10.1016/j.compbiomed.2021.104737 (CC BY-NC-ND 4.0) https://creativecommons.org/licenses/by/4.0/

Figure S6 below illustrates the example of patient images taken by Wang et al. [25] (2023) for the development of semi-supervised deep learning (SSL) model using MixMatch. There were nine photo positions taken in total (see Figure S6 (a)), although only five of them were selected for model training (see Figure S6b)).

Figure S6. *Examples of patients images taken from 9 different views.*

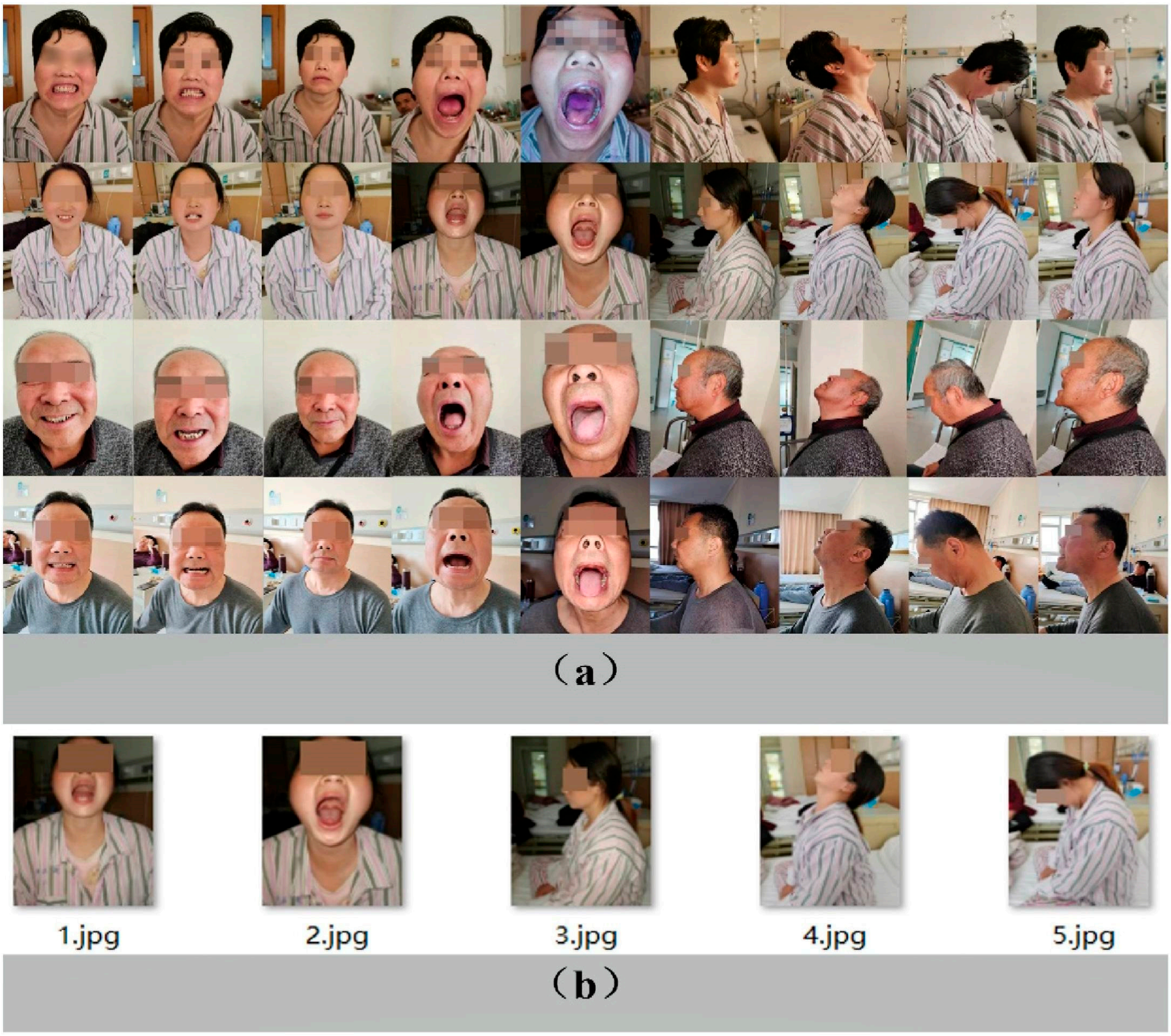

Note. (a) Examples of all 9 images of patients, with each row representing one patient's dataset. Areas around mouth and eyes blurred for confidentiality and accuracy enhancement. (b) Selection of five images from the total 9 images dataset (images 4-8). (Wang et al., 2023) [25].

Reproduced from © Wang, G.; Li, C.; Tang, F.; Wang, Y.; Wu, S.; Zhi, H.; Zhang, F.; Wang, M.; Zhang, J. A fully-automatic semi-supervised deep learning model for difficult airway assessment. Heliyon 2023, 9, e15629, doi:10.1016/j.heliyon.2023.e15629 (CC BY-NC-ND 4.0) https://creativecommons.org/licenses/by/4.0/

Figure S7 presents seven viewpoints, from which Xia et al [26] (2024) captured images of patients. However, only four of these positions were used in both facial and combined models (see Table S3A).

Figure S7. *Illustration of seven image positions.*

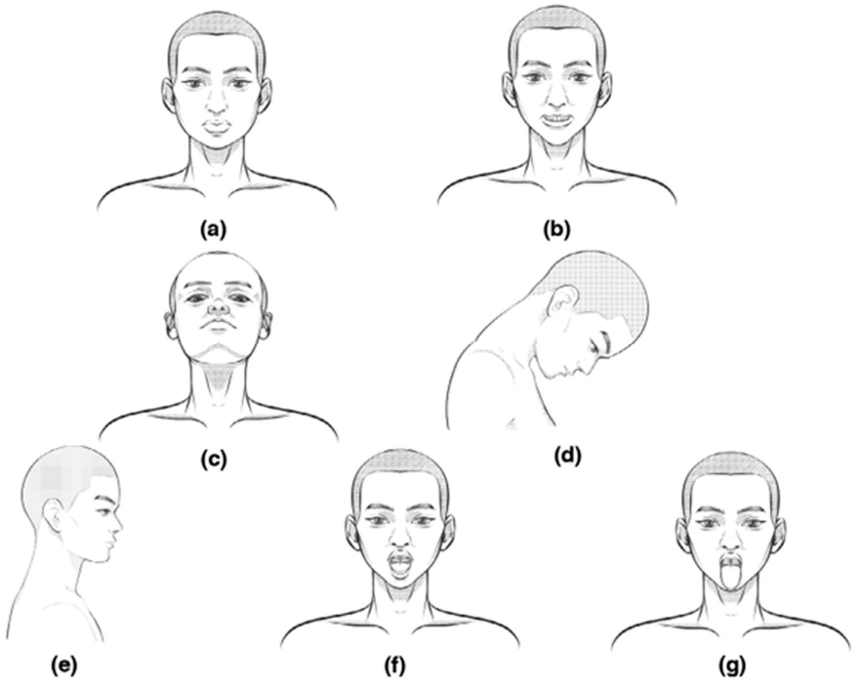

Note. The illustrations are seven positions of patients images taken for the research: (a) frontal neutral, (b) upper lip bite, (c) head-up, (d) head-down, (e) lateral, (f) open mouth, (g) extended tongue. (Xia et al., 2024) [26].

© Xia, M.; Jin, C.; Zheng, Y.; Wang, J.; Zhao, M.; Cao, S.; Xu, T.; Pei, B.; Irwin, M.G.; Lin, Z.; et al. Deep learning-based faci-al analysis for predicting difficult videolaryngoscopy: a feasibility study. Anaesthesia 2024, 79, 399-409, doi:10.1111/anae.16194 (CC BY-NC-ND 4.0) https://creativecommons.org/licenses/by/4.0/

Xia et al. [26] (2024) also used heatmaps for the determination of areas that could be important for difficult airway assessment (Figure S8). The left side heatmaps of each position were for patients with easy intubation, while the right ride was for difficult cases. This fact illustrates that red regions on heatmaps are necessary for difficult airway identification.

Figure S8. *Heat maps using gradient-weighted class activation maps (Grad-CAM++).*

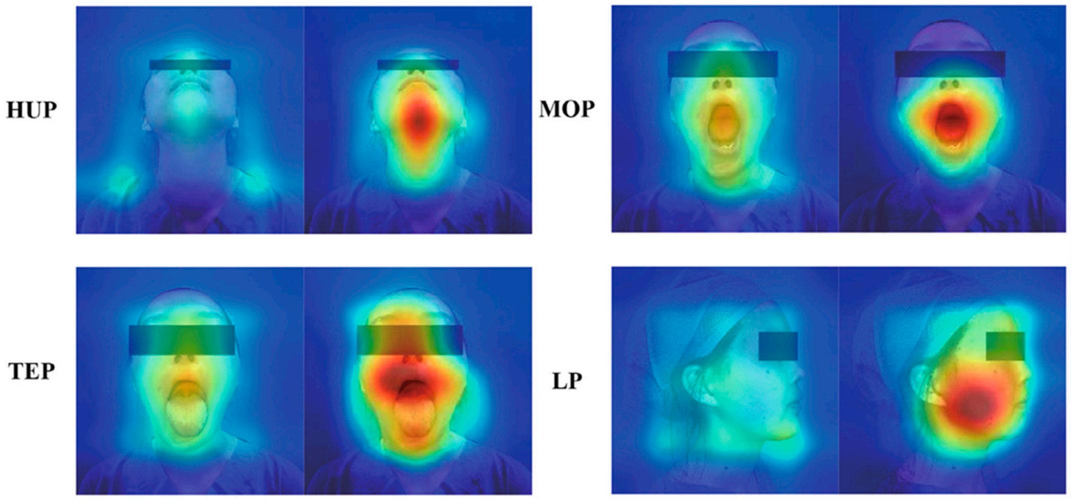

Note. The picture was aligned and divided in two for better alignment.

Note. The pictures are heatmaps of four positions of patient images: (HUP) head-up, (TEP) tongue extension, (MOP) open mouth, (LP) lateral. Patients with easy intubation cases on the left side, with difficult intubation cases on the right side. (Xia et al., 2024) [26].

© Xia, M.; Jin, C.; Zheng, Y.; Wang, J.; Zhao, M.; Cao, S.; Xu, T.; Pei, B.; Irwin, M.G.; Lin, Z.; et al. Deep learning-based faci-al analysis for predicting difficult videolaryngoscopy: a feasibility study. Anaesthesia 2024, 79, 399-409, doi:10.1111/anae.16194 (CC BY-NC-ND 4.0) https://creativecommons.org/licenses/by/4.0/

Figure S9 shows four photo views from which Kim et al. (2024) [22] took pictures of their patients. The four views did not necessarily limit the number of pictures for each patient, with some cases exceeding this number and taking additional photographs for better assessment and evaluation of airways by the predictive model.

Figure S9. *Patient photographs from four views for model training.*

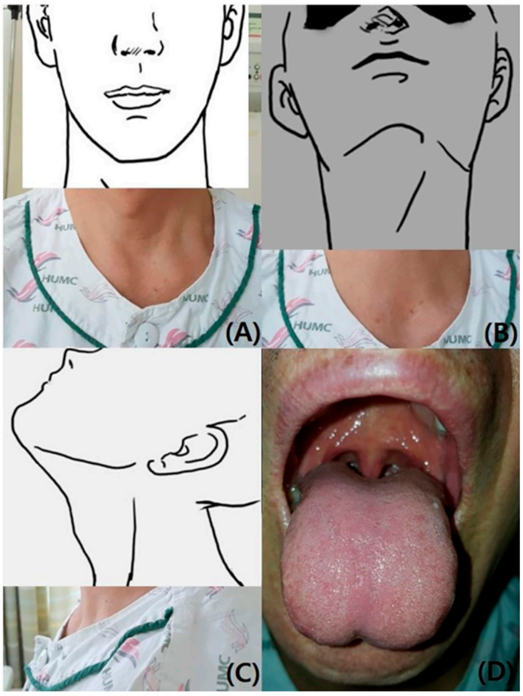

Note. Four image views are as follows: frontal view (A), frontal neck-extension view (B), lateral view (C), mouth-opening view (D). (Kim et al., 2024) [22].

© Kim, JH., Jung, HS., Lee, SE. et al. Improving difficult direct laryngoscopy prediction using deep learning and minimal image analysis: a single-center prospective study. Sci Rep 14, 14209 (2024). https://doi.org/10.1038/s41598-024-65060-x (CC BY-NC-ND 4.0)
https://creativecommons.org/licenses/by/4.0/

Additionally, Kim et al. [22] (2024) used heatmaps created with gradient-weighted class activation for the determination of the areas that help to predict difficult laryngoscopy (Figure S10).

Figure S10. *Gradient-weighted class activation mapping pictures (Grad-CAM).*

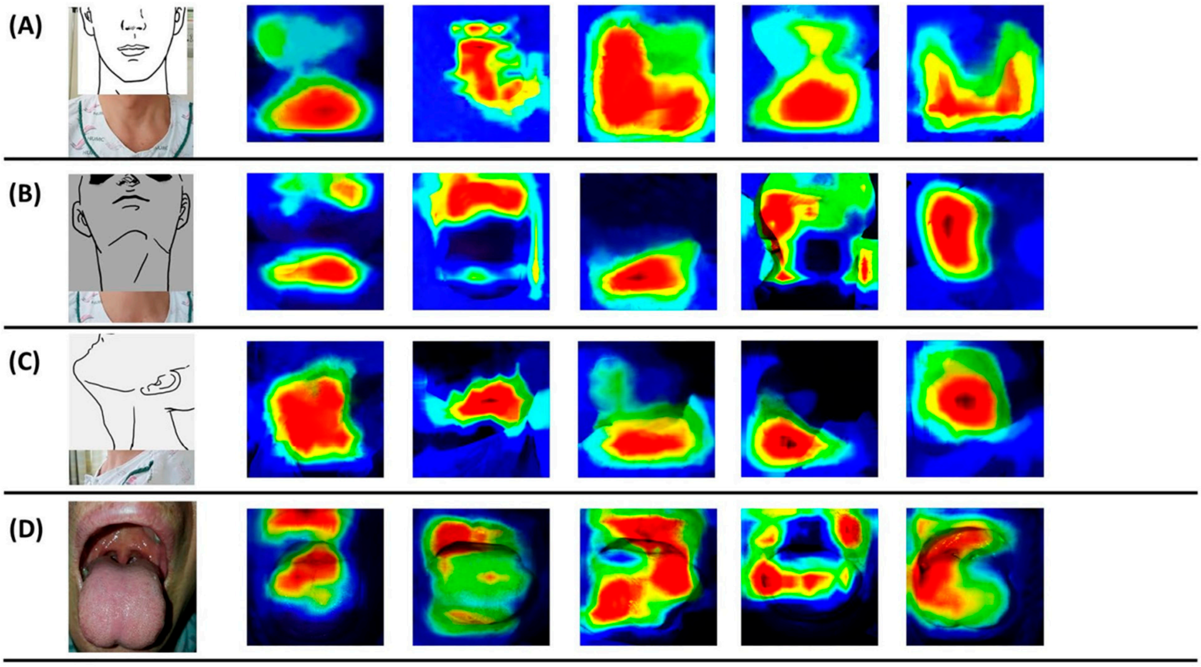

Note. The pictures in each row are of different patients for each of four viewpoints: frontal view (A), frontal neck-extension view (B), lateral view (C), mouth-opening view (D). (Kim et al., 2024) [22].

© Kim, JH., Jung, HS., Lee, SE. et al. Improving difficult direct laryngoscopy prediction using deep learning and minimal image analysis: a single-center prospective study. Sci Rep 14, 14209 (2024). https://doi.org/10.1038/s41598-024-65060-x (CC BY-NC-ND 4.0) https://creativecommons.org/licenses/by/4.0/

Supplementary Table S1. Database-specific search strategies for identifying AI/ML models for difficult airway prediction (search period: January 1, 2020 - July, 2025)

Database	Field(s) searched / Filters	Exact search string used*
PubMed	Fields: Title/Abstract + MeSH. Filters: Humans, English, 2020/01/01-2025/07/31	("Artificial Intelligence"[Mesh] OR "artificial intelligence"[tiab] OR "AI"[tiab] OR "machine learning"[tiab] OR "deep learning"[tiab]) AND ("difficult airway"[tiab] OR "difficult airway assessment"[tiab] OR "difficult intubation"[tiab] OR "airway management"[tiab]) AND ("2020/01/01"[Date - Publication] : "2025/07/31"[Date - Publication]) AND (humans[MeSH Terms]) AND (english[lang])
Web of Science Core Collection	Field: Topic (Title, Abstract, Author Keywords, Keywords Plus). Timespan: 2020-2025. Language: English	TS = (("artificial intelligence" OR "AI" OR "machine learning" OR "deep learning") AND ("difficult airway" OR "difficult airway assessment" OR "difficult intubation" OR "airway management")) AND LANGUAGE: (English) AND DOCUMENT TYPES: (Article OR Review) AND TIMESPAN: 2020-2025
Scopus	Fields: Title, Abstract, Keywords. Year: 2020-2025. Language: English	TITLE-ABS-KEY(("artificial intelligence" OR "AI" OR "machine learning" OR "deep learning") AND ("difficult airway" OR "difficult airway assessment" OR "difficult intubation" OR "airway management")) AND (LIMIT-TO(LANGUAGE, "English")) AND (PUBYEAR > 2019 AND PUBYEAR < 2025)

*Minor syntax adaptations were applied in each database to comply with platform-specific requirements (e.g., field tags, truncation, and date filters).

Supplementary Table S2. Core protocol for the systematic review of AI/ML models for predicting difficult airways

Domain	Item	Specification
**Research question**	Review objective	The goal is to systematically identify, evaluate, and synthesize studies that assess the diagnostic performance of artificial intelligence (AI) and machine learning (ML) models in predicting difficult airways/difficult intubation. Additionally, we aim to compare the performance of these models with that of conventional bedside clinical assessment tools.
**PICO framework**	Population	The study included patients undergoing airway assessment for tracheal intubation in clinical settings, such as the operating room, pre-anesthesia clinic, emergency department, and intensive care unit. There were no restrictions based on age, sex, or underlying disease.
	Index test	AI/ML-based models (including but not limited to deep learning, convolutional neural networks, machine learning classifiers, and hybrid models) using facial images and/or clinical variables to predict difficult airway / difficult intubation.
	Comparator	Traditional airway assessment methods and risk scores, such as the Mallampati classification, thyromental distance, the modified LEMON criteria, the upper lip bite test, and clinician judgment, were reported. Studies without explicit comparators were eligible if they reported the diagnostic performance of AI/ML models.
	Outcomes	Primary Outcome: Discriminative performance of AI/ML models for predicting a difficult airway/difficult intubation. This will be expressed as the area under the receiver operating characteristic curve (AUC). Where available, sensitivity and specificity will also be included. Secondary outcomes: Accuracy; positive and negative predictive values; calibration measures (e.g., calibration plots and Brier scores); decision curve analysis/net benefit; comparison of AI/ML versus traditional tests; and any reported clinical or workflow outcomes related to the use of the model.
**Eligibility criteria**	Inclusion criteria	(1) Original research articles or systematic/review articles that report primary data or synthesized evidence on AI/ML models for predicting difficult airways/difficult intubation; (2) Human participants who undergo airway assessment in a clinical care context; (3) An AI/ML model that is used as an index test to predict difficult airways/difficult intubation (with a binary or multiclass outcome); (4) At least one diagnostic performance metric that is reported for the AI/ML model (e.g., AUC, sensitivity, specificity, or accuracy); (5) Publication between January 1, 2020, and July 31, 2025; (6) Full text is available in English.
	Exclusion criteria	(1) Animal, cadaveric, simulation-manikin, or purely phantom studies; (2) Technical or methodological papers without an evaluation of a clinical or clinical image dataset; (3) Conference abstracts, letters, editorials, commentaries, or protocols without sufficient quantitative performance data; (4) Studies where the primary aim was not the prediction of a difficult airway/difficult intubation (e.g., airway anatomy segmentation without a

		predictive task); (5) Duplicate publications or overlapping datasets (in which case the most complete or recent report was included).
**Study designs**	Eligible designs	We included prospective or retrospective cohort studies, diagnostic accuracy studies, case-control studies, and systematic reviews that provided quantitative performance data for AI/ML airway prediction models.
**Setting and participants**	Clinical setting	Any hospital setting in which tracheal intubation is performed, including the operating room, preoperative assessment clinic, emergency department, and intensive care unit. There is no restriction on country or healthcare system.
**Information sources**	Databases	PubMed, Web of Science, Scopus
**Search strategy**	Keywords and operators	Core search concepts: (“artificial intelligence” OR “AI” OR “machine learning” OR “deep learning”) AND (“difficult airway” OR “difficult intubation” OR “airway assessment”). Boolean operators and database-specific controlled vocabulary (e.g., MeSH terms in PubMed) were applied as appropriate.
	Language restrictions	Two reviewers screened titles and abstracts independently against the eligibility criteria. Potentially relevant records were retrieved in full text and assessed for eligibility. Any disagreements were resolved through discussion and, if necessary, adjudication by a third reviewer.
**Data extraction**	Data items	The pre-specified items included: (1) bibliographic details (first author, year, and country); (2) study design and clinical setting; (3) sample size and the number and proportion of difficult airway cases; (4) population characteristics (age, sex, key comorbidities, and elective versus emergency); (5) the definition of "difficult airway" or "difficult intubation"; (6) the AI/ML model type and input features (e.g., facial images or clinical variables); (7) details of the training, validation, and test sets; (8) the presence and type of external validation; (9) comparator tests, if any; (10) performance metrics (AUC, accuracy, sensitivity, specificity, positive predictive value [PPV], negative predictive value [NPV], and calibration measures); (11) explainability techniques used, if any; and (12) reported implementation or clinical outcome data, if any.
	Extraction process	Two reviewers independently extracted the data using a standardized, piloted extraction form. Any discrepancies were resolved by consensus, and persistent disagreements were resolved by a third reviewer. Corresponding authors were contacted for missing key data, if necessary, although no formal imputation was planned.
**Risk of bias / quality assessment**	Tools	For original diagnostic accuracy studies, we assessed the risk of bias and applicability using a modified QUADAS-2 framework tailored to the evaluation of AI/ML models (including the following domains: patient selection, index test, reference standard, flow and timing, and AI-specific items, such as dataset partitioning and external validation). For review articles, the

		methodological quality was assessed descriptively (e.g., clarity of the search strategy, inclusion criteria, and synthesis methods).
**Synthesis and analysis plan**	Primary synthesis method	Due to the expected heterogeneity in model architectures, input features, outcome definitions, and performance metrics, the primary synthesis was designed as a structured narrative synthesis. Studies were grouped according to the following criteria: type of AI/ML approach (e.g., deep learning versus traditional machine learning), input data (image-based versus combined clinical and imaging data), and validation strategy (internal versus external).
	Planned comparisons	(1) A comparison of the performance of AI/ML models with that of traditional bedside tests (e.g., the Mallampati test, the thyromental distance test, and the modified LEMON test) when reported in the same cohort; (2) a comparison of the performance of internal and external validation datasets; and (3) a descriptive comparison of image-only models versus multimodal models (image + clinical variables).
	Subgroup and sensitivity analyses	Where data permitted, the following planned subgroup/sensitivity analyses were included: (1) elective versus emergency settings, (2) adult versus pediatric cohorts, (3) presence versus absence of external validation, and (4) studies at lower versus higher risk of bias according to QUADAS-2. Due to limited and heterogeneous data, these analyses were primarily descriptive.
**Deviations from protocol**	Recording and reporting	Any deviations from the pre-specified protocol, such as an inability to conduct a meta-analysis or a lack of data for planned subgroups, were documented and reported in the Discussion and Limitations sections. No unplanned outcomes were added post hoc.

Supplementary Table S3A. Comparison of original research papers in terms of participants, data and existing methods.

Criteria		Hayasaka et al. (2021) [7]	Tavolara et al. (2021) [24]	Wang et al. (2023) [25]	Xia et al. (2024) [26]
Participants	Number	202 (92 male, 110 female).	505<br>(76 difficult to intubate, 429 easy to intubate).	1000<br>(455 male, 545 female; 242 difficult airway, 758 normal airway patients)	5849 (5335 non-difficult, 514 difficult videolaryngoscopy cases)
	Ethnicity	Japanese (Yamagata University Hospital)	Not indicated	Chinese (Henan Provincial People's Hospital, Henan, China).	Chinese, 98.6% of total participants (data taken from the table with patients characteristics).<br><br>The nationality of the rest were not indicated.
	Criteria	Initial total: 1043<br><br>Excluded: 838.<br><br>**For exclusion:**<br><br>1. Were younger than 20;<br>2. Were intubated by a physician with <3 years experience;<br>3. Were not intubated by a Macintosh laryngoscope (during initial intubation);<br>4. Had surgery altering facial appearance (nasal surgery, ophthalmology, dentistry, neurosurgery, heart surgery);<br>5. Had surgery that changed neck movement range (esophageal, cervical spine, thyroid surgeries);<br>6. Were intubated	**For cross-validation:**<br><br>All difficult cases, and randomly picked set of 76 easy to intubate patients	**For inclusion:**<br><br>1. Older than 18;<br>2. Had elective surgery under general anesthesia;<br>3. Had grade 1-3 physical status classification (by ASA[a]);<br>4. Signed informed consent form.<br><br>**For exclusion:**<br><br>1. Had head, neck, oral or maxillofacial deformities, absence of incisors, subglottic stenosis, severe maxillofacial trauma or cervical spondylosis;<br>2. Had psychiatric disorders or central nervous system diseases;<br>3. Had communication	Initial total: 7194<br><br>Excluded: 1345<br><br>**For exclusion:**<br>1. Had incomplete images;<br>2. Were without tracheal intubation data.

Criteria		Hayasaka et al. (2021) [7]	Tavolara et al. (2021) [24]	Wang et al. (2023) [25]	Xia et al. (2024) [26]
		by other devices;<br>7. Had dementia or psychiatric disorders;<br>8. Were managed with supraglottic airway devices;<br>9. Participated in other studies.		disorders, such as language barrier, severe visual or hearing impairment.	
Data	Type	Patient face images from 16 positions (see Figure S1)*.<br>Baseline characteristics:<br>• Age (Mean = 63.9 ± 14.2 years),<br>• Sex,<br>• BMI,<br>• Comorbidities,<br>• MP[b],<br>• TMD[b],<br>• IIG[b],<br>• HNM[b],<br>• HLM[b],<br>• BT[b],<br>• ULBT.<br>Class activation heatmaps (see Figures 3 and 4).<br>ASA Physical status (PS):<br>• PS 1 – 15.8%;<br>• PS 2 – 67.8%;<br>• PS 3 – 16.3%.<br>Average clinical experience of anesthesiologists: 11.2 ± 6.9 years.	Celebrity face images (CASIA-Webface);<br>Frontal face images (patients);<br>MP, TMD.	9 images: 5 frontal, 4 side views (see Figure S6 (a)).<br>5 images selected for research from different angles (see Figure S6 (b)).<br>Mean age: 62.8 years<br>Mean BMI: 23.5<br>ASA Physical status (PS):<br>• PS 1 – 14.3%;<br>• PS 2 – 69.3%;<br>• PS 3 – 16.4%.<br>6 airway difficulty predictors:<br>• mandibular mobility,<br>• maximum mouth opening,<br>• TMD,<br>• MP,<br>• neck movements,<br>• chin-to-chest distance.	Patient face images in 7 views: frontal neutral, upper lip bite, head-up, head-down, lateral, open mouth, extended tongue (see Figure S7).<br>Heat maps built using gradient-weighted class activation maps (Grad-CAM++) (see Figure S8). Include positions: head-up, tongue extended, mouth open, lateral.<br>Baseline characteristics:<br>• Age,<br>• Sex,<br>• Education,<br>• BMI,<br>• Alcohol consumption,<br>• Smoking,<br>• ASA PS.
	Processing	**Preprocessing:**	**Preprocessing** (see Figure S4)**:**	**Preprocessing:**	**Preprocessing:**

Criteria		Hayasaka et al. (2021) [7]	Tavolara et al. (2021) [24]	Wang et al. (2023) [25]	Xia et al. (2024) [26]
	and<br>Procedure	• Dataset divided into 8:2 ratio for training and test for inference evaluation.<br>• Training samples were expanded from 0.7 to 1.3 times to avoid overfitting (354 easy, 387 difficult cases).<br>• Bias between easy and difficult to intubate groups cleared.<br>• Images were resized for excess features and computational complexity reduction.<br>**Procedure:**<br>**CL[c] classification of patients:**<br>• General anesthesia induction;<br>• Tracheal intubation with Macintosh laryngoscope;<br>• CL classification documented in medical records (grades I-IV), grades III-IV labeled as difficult to intubate.<br>**Two deep learning methods:**<br>*Transfer learning:*<br>• Incorporate trained model VGG16 into to-be-	• CASIA- Webface (dataset divided for training, validation and testing in 85:5:10 ratio).<br>• Landmark detection: 68 landmarks on face, including eyes, nose, mouse, lips, chin, cheeks, eyebrows; custom neck landmarks.<br>• Face alignment done with OpenFace. Three sets of points for alignment: inner corner of eyes and bottom center of lips, outer corner of eyes and bottom of nose, no alignment.<br>• Face Region Feature Extractor (FRFE). Grayscale, 100x100 images cropped while centered on specific 11 landmarks.<br>**Processing:**<br>• Labeling a collection of images (bags).<br>• Each bag with 88 vector features extracted using FRFE.<br>**Procedure:**<br>Two strategies:<br>• Retraining the last layer of FRFE	• Images dataset divided into 8:2 ratio for training and testing.<br>• 30% of training samples were labeled.<br>• Data augmentation for increasing the number of normal and difficult airway cases by factors of 2 and 6, respectively, to create balance.<br>**Procedure:**<br>**CL[c] classification of patients:**<br>• Routine monitoring: ECG[d], NBP[d], SpO_2[d], $EtCO_2$[d];<br>• Anesthesia induction: midazolam (0.03 mg/kg), sufentanil (0.5-1.0 μg/kg), etomidate (0.3-0.6 mg/kg), rocuronium (0.6-0.9 mg/kg);<br>• Mask ventilation;<br>• CL classification (grades I-IV), grades III-IV labeled as difficult to intubate.<br>• No certain CL classification labeled as difficult.<br>• Manual labeling of images as difficult/normal airways by three anesthesiologists	• Dataset of images randomly split in ratios of 6:2:2 for training, validation, and testing.<br>• Random duplication of difficult samples until they reached the same number of non-difficult cases was done to solve the imbalance issue.<br>**Procedure:**<br>**CL[c] classification of patients:**<br>• Routine monitoring: pulse oximetry, ECG, NBP;<br>• Anesthesia induction: midazolam (2-3 mg), propofol (2-3 mg/kg), fentanyl (2-4 μg/kg), rocuronium (0.6 mg/kg);<br>• Mask ventilation;<br>• CL classification (grades I-IV), grades III-IV labeled as difficult to intubate.<br>• Tracheal intubation with videolaryngoscop y by one anesthesiologist;<br>• Two other independent anesthesiologists completed CL assessment, with a third senior making the final

Criteria	Hayasaka et al. (2021) [7]	Tavolara et al. (2021) [24]	Wang et al. (2023) [25]	Xia et al. (2024) [26]
	created model;<br>• Trained from 14 million images;<br>• Composed of 16 layers (13 convolutional and 3 fully connected layers).<br>*Fine tuning:*<br>• Added additional layer to VGG16 model's 13 convolutional layers;<br>• Classifies final output as easy or difficult according to input picture.	model, summing the outcome of all rotations (all 88);<br>• Apply attention-based MIL[e] to FRFE extractors (see Figure S5).	(>5 years of clinical experience):<br>• Multi-channel fusion of information, accompanied with MixMatch SSL and ResNet18 backbone network.	decision.<br>**Procedure:**<br>• LASSO logistic regression and 10-fold cross-validation.
Performance evaluation	**ULBT:**<br>• AUC 0.70.<br>**MP:**<br>• AUC 0.60.<br>**Modified LEMON criteria:**<br>• Sensitivity 85%, specificity 47%.<br>**Compared with 16 AI models:**<br>**MP scores:**<br>• Sensitivity 79.6%, specificity 52.7%, AUC 0.673.	**Compared with last layer replaced FRFE model:**<br>**MP scores:**<br>• Sensitivity 35.53, specificity 78.95, AUC 0.5748.<br>**TMD scores:**<br>• Sensitivity 88.16, specificity 3.95, AUC 0.4933.<br>**Compared with MIL model:**<br>**MP scores:**<br>• Sensitivity 81.58, specificity 35.53, AUC 0.6042.<br>**TMD scores:**<br>• Sensitivity 88.16, specificity 10.53, AUC 0.4661.<br>**One-sided McNemar's test, MIL model for:**<br>**MP scores:**	**Ground truth value, three doctors results:**<br>• Accuracy 91.00%, sensitivity 91.67%, specificity 90.79%, F1 83.26%, AUC 0.9497.	The tongue extension, mouth open and **ULBT** positions: AUROC > 0.7<br>The **combined model**: AUROC of 0.778<br>The **facial model**: AUROC of 0.779

Criteria		Hayasaka et al. (2021) [7]	Tavolara et al. (2021) [24]	Wang et al. (2023) [25]	Xia et al. (2024) [26]
			• p = 0.0151.<br><br>**TMD scores:**<br>• p = 0.0001.		

a ASA, American Society of Anesthesiologists.
b MP, Mallampati test; TMD, thyromental distance; IIG, inter-incisior gap; HNM, head and neck movements; HLM, horizontal length of mandible; BT, buck teeth; ULBT, upper lip bite test.
c CL, Cormack-Lehane classification.
d ECG, electrocardiogram; NBP, non-invasive blood pressure; SpO2, oxygen saturation level; EtCO2, end-tidal carbon dioxide concentration.
e MIL, Multiple instance learning.
* All figures mentioned hereafter are placed separately in the Supplemental Materials.

Supplementary Table S3B. Comparison of original research papers in terms of participants, data and existing methods.

Criteria		Kim et al. (2024) [22]	Kim et al. (2021) [23]	Yamanaka et al. (2022) [27]
Participants	Number	3053<br><br>1283 (after under-sampling): 771 NDDL[a], 512 DDL[a]	1677<br>(1467 NDL[d], 210 DL[d]).	10741<br>(543 difficult airways, 7690 first-pass success)
	Ethnicity	Korean (Sacred Heart Hospital in Chuncheon, South Korea)	Korean (Hallym University Chuncheon Sacred Heart Hospital)	Japanese (13 academic and 1 community emergency departments across Japan)
	Criteria	Initial total: 3305<br>Excluded: 252<br><br>**For inclusion:**<br>1. Were older than 18;<br>2. Were scheduled for endotracheal intubation under general anesthesia;<br>3. Were in the cervical spine surgery or some emergency surgeries.<br><br>**For exclusion:**<br>1. Had errors or incomplete records;<br>2. Were intubated outside the operating room;	Initial total: 7765<br>Excluded: 6088<br><br>**For exclusion:**<br>1. Were under 18;<br>2. Had regional anesthesia;<br>3. Had major external facial or neck abnormalities;<br>4. Had laryngeal abnormalities or tumors;<br>5. Used laryngeal mask;<br>6. Mask ventilation only;<br>7. Used video laryngoscope;<br>8. Used fiberoptic scope;	Initial total: 10816<br>Excluded: 75<br><br>**For exclusion:**<br>1. Had surgical intubation at first attempt

Criteria		Kim et al. (2024) [22]	Kim et al. (2021) [23]	Yamanaka et al. (2022) [27]
		3. Unconscious;<br>4. Had major external facial or neck abnormalities;<br>5. Had laryngeal abnormalities or tumors;<br>6. Had full stomachs.	9. Missing data;<br>10. Stated endotracheal intubation or tracheostomy before anesthesia.	
Data	Type	Patient face images from 4 views (see Figure S9).<br><br>Baseline characteristics:<br>● Age: 58 (IQR[b] 45-68),<br>● Sex: 1681 males (55.1%),<br>● BMI: 24.8 (IQR 22.6-27.3),<br>● Height, cm: 163 (IQR 155.9-170)<br>● Weight, kg: 65.8 (IQR 57.3-74.7)<br>● Emergency surgery: 107 (3.5%)<br><br>Class activation heatmaps (see Figures S10).<br><br>Clinical practitioners: 13<br>The classification of their experience:<br>● 1-2 years<br>● 2-3 years<br>● 3-4 years<br>● 4-5 years<br>● over 5 years	Neck circumference, Thyromental height.<br><br>DL predictors for NDL (n=1467):<br>● Age: 57 (IQR[b] 43-66),<br>● Sex: 793 males (54.1%),<br>● BMI: 24.9 (IQR 22.8-27.4),<br>● Height, cm: 162.7(IQR 156.1-169.7)<br>● Weight, kg: 66.5 (IQR 57.8-75.0)<br>● NC[e], cm: 36.8 (IQR 34.1-39.2)<br>● TMHT[e], cm: 5.5 (IQR 4.7-6.4)<br><br>DL predictors for DL (n=210):<br>● Age: 61 (IQR[b] 49-68),<br>● Sex: 132 males (62.9%),<br>● BMI: 25.1 (IQR 23.2-27.3),<br>● Height, cm: 164.9 (IQR 155.9-170)<br>● Weight, kg: 66.8 (IQR 59.0-78.8)<br>● NC[e], cm: 37.8 (IQR 35.4-40.0)<br>● TMHT[e], cm: 5.4 (IQR 4.4-6.2)	Airway management details:<br>● Pre-intubation vital signs (systolic blood pressure, pulse rate, respiratory rate, oxygen saturation);<br>● Glasgow coma scale;<br>● Modified LEMON criteria;<br>● Week day;<br>● Medications;<br>● Intubation methods, devices;<br>● Intubators' year of study, specialty.<br><br>Baseline characteristics:<br>● Age: 71 (IQR 56-81);<br>● Children: 304 (2.8%);<br>● Sex: 4079 females (38.0%);<br>● Height, cm: 160 (IQR 153-170);<br>● Weight, kg: 60 (IQR 50-67);<br>● BMI: 22.0 (IQR 19.5-24.3).<br><br>Intubation outcomes:<br>● Difficult airways: 543 (5.1%);<br>● First-pass success: 7690 (71.6%);<br>● Aborted intubation attempts: 39
	Processing and Procedure	**Preprocessing:**<br>● Modification of the base network for addition of subtask to the main task	**Preprocessing:**<br>● Dataset divided for training and testing sets in	**Preprocessing:**<br>● Dataset divided into 8:2 ratio for training and

Criteria	Kim et al. (2024) [22]	Kim et al. (2021) [23]	Yamanaka et al. (2022) [27]
	about clinical experience of physicians.<br>• Pictures not limited to four, some additional taken for better evaluation of airways on the day before or of surgery.<br>• Under-sampling of pictures to match DDL and NDDL cases in 1:1 ratio.<br><br>**Procedure:**<br>**CL[c] classification of patients:**<br>• Muscle relaxation: rocuronium (0.6-0.8 mg/kg);<br>• Standardized depth of anesthesia induction: propofol with additional opioids;<br>• CL classification documented in medical records (grades I-IV), grades III-IV labeled as DDL:<br>• Sometimes external laryngeal manipulation applied to improve laryngeal view and help in intubation;<br>• Tracheal intubation with standard Macintosh metallic single-use disposable laryngoscope blade.<br>• Supine position, using standard sized pillow<br>• Fourfold cross-validation in training.	80:20 ratio.<br>• Each set had the same ratio of NDL and DL cases.<br>• DL cases oversampling through SMOTE[f].<br>• Training of all sets with five algorithms.<br><br>**Procedure:**<br>Tracheal intubation:<br>• Completed by seven attending anesthesiologists and five resident anesthesiologists.<br>• Use of standard Macintosh metallic single-use disposable laryngoscope blades for intubation.<br>• Direct laryngoscopies classified according to CL classification, with grades 3-4 considered as DL.	testing.<br>• Imputations for missing variables using random forest.<br>• Predictor preprocessing, one-hot encoding, normalization, standardization.<br><br>**Procedure:**<br>• In training, 7 machine learning algorithms models developed.<br>• Reference for difficult airways outcome: modified LEMON criteria.<br>• Reference for first-pass success: logistic regression.<br>• Five-fold cross-validation for identification of hyperparameters with the highest c-statistic.<br>• In testing, comparisons between models and references.
Performance evaluation	AUROC 0.82–0.86 and sensitivity 0.63–0.9<br>F1-score of 0.72–0.81 for DDL prediction.	**Neck circumference used as single predictor:**<br>• AUROC 0.57<br><br>**Thyromental height used as**	**Modified LEMON criteria:**<br>• Sensitivity 0.67, specificity 0.57, PPV[g] 0.08, NPV[g] 0.97,

Criteria	Kim et al. (2024) [22]	Kim et al. (2021) [23]	Yamanaka et al. (2022) [27]
		**single predictor:**<br>• AUROC 0.45.	PLR[g] 1.57, NLR[g] 0.57.<br>**Logistic regression:**<br>• Sensitivity 0.91, specificity 0.36, PPV 0.78, NPV 0.61, PLR 1.42, NLR 0.26.

a NDDL, non-difficult direct laryngoscopy; DDL, difficult direct laryngoscopy.
b IQR, interquartile range.
c CL, Cormack-Lehane classification.
d NDL, non-difficult laryngoscopy; DL, difficult laryngoscopy.
e NC, neck circumference; TMHT, thyromental height.
f SMOTE, synthetic minority oversampling technique.
g PPV, positive predictive value; NPV, negative predictive value; PLR, positive likelihood ratio; NLR, negative likelihood ratio.
Note. NC was considered as a circumference on the level of thyroid cartilage; TMHT was defined as height between the anterior border of the thyroid cartilage and the anterior border of the mentum.
